# Recent applications of EEG-based brain-computer-interface in the medical field

**DOI:** 10.1186/s40779-025-00598-z

**Published:** 2025-03-24

**Authors:** Xiu-Yun Liu, Wen-Long Wang, Miao Liu, Ming-Yi Chen, Tânia Pereira, Desta Yakob Doda, Yu-Feng Ke, Shou-Yan Wang, Dong Wen, Xiao-Guang Tong, Wei-Guang Li, Yi Yang, Xiao-Di Han, Yu-Lin Sun, Xin Song, Cong-Ying Hao, Zi-Hua Zhang, Xin-Yang Liu, Chun-Yang Li, Rui Peng, Xiao-Xin Song, Abi Yasi, Mei-Jun Pang, Kuo Zhang, Run-Nan He, Le Wu, Shu-Geng Chen, Wen-Jin Chen, Yan-Gong Chao, Cheng-Gong Hu, Heng Zhang, Min Zhou, Kun Wang, Peng-Fei Liu, Chen Chen, Xin-Yi Geng, Yun Qin, Dong-Rui Gao, En-Ming Song, Long-Long Cheng, Xun Chen, Dong Ming

**Affiliations:** 1 State Key Laboratory of Advanced Medical Materials and Devices, Medical School, Tianjin University, Tianjin, 300072 China; 2 Haihe Laboratory of Brain-Computer Interaction and Human-Machine Integration, Tianjin, 300380 China; 3 School of Pharmaceutical Science and Technology, Tianjin University, Tianjin, 300072 China; 4 Department of Micro/Nano Electronics, Shanghai Jiaotong University, Shanghai, 200240 China; 5 Institute for Systems and Computer Engineering, Technology and Science, 4099-002 Porto, Portugal; 6 Institute of Science and Technology for Brain-Inspired Intelligence, Fudan University, Shanghai, 200433 China; 7 School of Intelligence Science and Technology, University of Sciences and Technology Beijing, Beijing, 100083 China; 8 Tianjin Huanhu Hospital, Tianjin, 3003500 China; 9 The State Key Laboratory of Brain and Cognitive Sciences, The University of Hong Kong, Hong Kong SAR, 999077 China; 10 State Key Laboratory for Quality Ensurance and Sustainable Use of Dao-Di Herbs, Artemisinin Research Center, and Institute of Chinese Materia Medica, China Academy of Chinese Medical Sciences, Beijing, 100700 China; 11 Department of Neurosurgery, Beijing Tiantan Hospital, Capital Medical University, Beijing, 100070 China; 12 China National Clinical Research Center for Neurological Diseases, Beijing, 100070 China; 13 Medical Research Council Brain Network Dynamics Unit, Nuffield Department of Clinical Neurosciences, University of Oxford, Oxford, OX1 3TH UK; 14 Department of Electric Engineering and Information Science, University of Science and Technology of China, Hefei, 230026 China; 15 Department of Rehabilitation, Huashan Hospital, Fudan University, Shanghai, 200040 China; 16 Xuanwu Hospital of Capital Medical University, Beijing, 100053 China; 17 The First Hospital of Tsinghua University, Beijing, 100016 China; 18 Department of Critical Care Medicine, West China Hospital of Sichuan University, Chengdu, 610041 China; 19 Department of Neurosurgery, The First Hospital of China Medical University, Beijing, 110122 China; 20 Department of Critical Care Medicine, Division of Life Sciences and Medicine, The First Affiliated Hospital of University of Science and Technology of China, University of Science and Technology of China, Hefei, 230031 China; 21 School of Computer Science, Fudan University, Shanghai, 200438 China; 22 School of Life Science and Technology, University of Electronic Science and Technology of China, Chengdu, 611731 China; 23 Shanghai Frontiers Science Research Base of Intelligent Optoelectronics and Perception, Institute of Optoelectronics, Fudan University, Shanghai, 200433 China

**Keywords:** Brain-computer interfaces (BCIs), Medical applications, Rehabilitation, Communication, Brain monitoring, Diagnosis

## Abstract

Brain-computer interfaces (BCIs) represent an emerging technology that facilitates direct communication between the brain and external devices. In recent years, numerous review articles have explored various aspects of BCIs, including their fundamental principles, technical advancements, and applications in specific domains. However, these reviews often focus on signal processing, hardware development, or limited applications such as motor rehabilitation or communication. This paper aims to offer a comprehensive review of recent electroencephalogram (EEG)-based BCI applications in the medical field across 8 critical areas, encompassing rehabilitation, daily communication, epilepsy, cerebral resuscitation, sleep, neurodegenerative diseases, anesthesiology, and emotion recognition. Moreover, the current challenges and future trends of BCIs were also discussed, including personal privacy and ethical concerns, network security vulnerabilities, safety issues, and biocompatibility.

## Background

Brain-computer interfaces (BCIs) are advanced communication systems that leverage brain activity signals as a medium, converting them into desired outputs to enable users to operate external devices through brain activity without relying on peripheral nerve or muscle control. BCIs can be categorized into invasive and non-invasive [1]. Invasive BCIs involve surgically implanting electrodes directly into the cerebral cortex, such as electrocorticography (ECoG) and local field potentials, to obtain high-quality and high-resolution neural signals that accurately capture the details of neural activity [2]. This approach provides continuous and stable signal recordings suitable for long-term monitoring and control applications [3]. Non-invasive BCIs record brain activity using external devices such as electroencephalogram (EEG), functional near-infrared spectroscopy (fNIRS), functional magnetic resonance imaging (fMRI), and magnetoencephalography (MEG). The advantages and disadvantages of these methods are summarized in Table 1. Among non-invasive technologies, EEG signals are particularly well-suited for practical applications in real-life scenarios due to their millisecond-level high temporal resolution, which makes them ideal for capturing rapid changes in brain activity. Moreover, EEG equipment is cost-effective, highly portable, and widely used, making it invaluable in both research and clinical settings. Consequently, EEG signals are extensively preferred in the field of BCIs.

**Table 1:** Types of signals for BCI-related technologies

Signal type	Acquisition method	Resolution (spatial/temporal)	Invasiveness	Primary usage in BCIs	Key advantages	Key disadvantages
EEG	Scalp electrodes	Low spatial; High temporal	Non-invasive	Widely used	Portable; Low cost; High temporal resolution	Poor spatial resolution; Sensitive to artifacts
MEG	Magnetic field sensors	High spatial; High temporal	Non-invasive	Research; Less common	High spatial and temporal resolution	Expensive; Bulky; Requires shielding
fMRI	Magnetic resonance	High spatial; Low temporal	Non-invasive	Research; Rare	Excellent spatial resolution	Low temporal resolution; Expensive; Slow
fNIRS	Near-infrared light	Low spatial; Low temporal	Non-invasive	Research; Growing use	Portable; Safe; Can monitor over time	Low resolution; Limited to cortical signals
ECoG	Cortical surface electrodes	High spatial; High temporal	Semi-invasive	Research; Experimental	High spatial and temporal resolution; Less noise	Invasive; Risk of infection; Surgical implantation required
LFPs	Deep brain electrodes	High spatial; High temporal	Invasive	Research; Experimental	Good resolution; Detect deep brain signals	Invasive; Surgical risks; Used for specific applications

*EEG* electroencephalogram, *MEG* magnetoencephalography, *fMRI* functional magnetic resonance imaging, *fNIRS* functional near-infrared spectroscopy, *ECoG* electrocorticography, *LFPs* local field potentials, *BCI* brain-computer interface

Advancements in neuroscience, engineering, and computational methods have significantly propelled the evolution of BCI technology. As technology continues to progress, there is a growing trend toward non-invasive approaches. Invasive BCIs, which require surgical implantation of electrodes into the brain, offer the most direct and accurate access to neural signals [4]. They provide highly precise neural signals with minimal interference from surrounding tissue or the skull [5]. Moreover, they support higher bandwidth, facilitating real-time control and communication. However, invasive BCIs pose significant safety risks, such as infection, and electrode performance may deteriorate over time due to tissue responses like scarring, which can reduce the fidelity of signal transmission [6]. Therefore, researchers are increasingly exploring less risky alternatives. The shift towards non-invasive BCIs is driven by several key factors. Firstly, advancements in feature extraction and signal preprocessing algorithms have significantly enhanced the ability to extract meaningful information from low signal-to-noise ratio and low-resolution signals [7]. Secondly, the development of portable, wearable BCI systems enables their use in real-world environments beyond laboratory settings [8]. Thirdly, users generally prefer non-invasive devices for daily or rehabilitative purposes, as these options avoid the risks and concerns associated with brain surgery. Lastly, there is growing interest in applying non-invasive BCIs in areas such as gaming, health, and education, where practicality and accessibility are paramount.

BCIs have always been a hot field of interest for researchers. Numerous researchers have dedicated efforts to designing diverse human–computer communication modes. This field has evolved from an initial concept into a practical technology that integrates signal recognition, recording, and analysis [9]. In 1929, Berger [10] first recorded an EEG from the scalp of a boy with a brain tumor, providing early insights into neural activities [11]. Since then, EEG signals have been widely used in clinical settings to diagnose brain disorders. In 1973, Vidal [12] pioneered the use of EEG for human–computer interaction and introduced the term “BCI”. With advancements in neuroscience and engineering, BCIs are now entering a new era of broad applications.

Modern BCIs are artificial intelligence (AI)-based systems that process brain activity in real-time to recognize specific central nervous system (CNS) activity patterns. A typical BCI system usually consists of 5 consecutive stages: signal acquisition, preprocessing or signal enhancement, feature extraction, classification, and control interface [13]. First, during the signal acquisition phase, the BCI systems capture raw neural signals using various sensors, such as EEG electrodes, ECoG electrodes, and fNIRS sensors. Second, in the preprocessing stage, the aim is to improve signal quality in subsequent analyses. Standard preprocessing techniques include filtering, denoising, and normalization to remove interference and noise, thereby improving signal reliability and usability. Third, feature extraction involves extracting representative information from the preprocessed signal. Fourth, machine learning or deep learning (DL) algorithms map the extracted features to predefined categories or states. This stage involves training and applying classification models to achieve accurate pattern recognition and classification. Finally, the classification results are converted into specific control commands to drive external devices or applications [14].

BCIs play a crucial role in modern science and engineering, with applications spanning both healthy individuals and clinical patients [15]. BCI technology offers augmented reality experiences, brainwave-controlled devices, and novel functionalities for healthy populations, enhancing learning and productivity. Moreover, BCIs are particularly significant in the medical field, especially for patients suffering from severe neurological diseases and injuries [16]. For example, individuals with locked-in syndrome (LIS) retain full cognitive awareness but are unable to communicate or move voluntarily. Conditions such as amyotrophic lateral sclerosis (ALS) [17], cerebral palsy [18, 19], brainstem stroke [20], multiple sclerosis [21], and spinal cord injury (SCI) [22] are leading causes of LIS. BCI technology provides these individuals with a means to communicate at a basic level, significantly improving their quality of life. Furthermore, integrating BCIs with rehabilitation equipment can create an active closed-loop rehabilitation system for clinical patients [23]. These systems facilitate more effective recovery by providing real-time monitoring and feedback during rehabilitation. Additionally, BCI technology is vital in diagnosing and treating Alzheimer’s disease (AD) and Parkinson’s disease (PD), detecting and intervening in sleep disorders, assessing consciousness levels, and monitoring anesthesia depth.

In recent years, several review articles have examined various aspects of BCIs, including their foundational principles, technical advancements [24], and applications in specific domains [25]. For example, Chaddad et al. [26] provided a comprehensive analysis of signal processing techniques in EEG-based BCI systems, with a focus on feature extraction and classification methods. Similarly, Saibene et al. [27] reviewed advances in wearable BCIs, highlighting the development of portable and user-friendly systems. Other reviews, such as those by Zhuang et al. [28], emphasized the role of invasive BCIs in neurorehabilitation and motor control. While these studies have significantly enhanced our understanding of specific aspects of BCIs, there remains a notable gap in the literature regarding systematic explorations of BCI applications across a broad range of medical fields. Additionally, few studies have addressed the interdisciplinary challenges and future directions necessary for advancing clinical applications.

This review aims to provide an extensive overview of recent applications of EEG-based BCIs in the medical field across 8 critical areas, including rehabilitation, daily communication, epilepsy, cerebral resuscitation, sleep, neurodegenerative diseases, anesthesiology, and emotion recognition. Unlike previous reviews that focus on specific technologies or limited applications, this paper integrates insights from both invasive and non-invasive BCIs, emphasizing their clinical relevance and transformative potential in healthcare. Furthermore, this review identifies key technical, ethical, and privacy challenges that BCIs face in bedside applications, offering a roadmap for future research and development.

## BCI signal processing techniques

The basic process of the BCIs includes signal acquisition, preprocessing, feature extraction, classification, and task application. Consequently, given the use of identical EEG acquisition devices, the effectiveness of BCIs mainly depends on the quality of preprocessing and feature extraction. This section will focus on these two key aspects.

### Preprocessing

Preprocessing is an important step in the analysis of EEG data, aiming to remove noise, artifacts, and irrelevant information, thus facilitating subsequent analysis. The preprocessing of EEG signals generally encompasses downsampling, artifact removal, and feature scaling.

#### Downsampling

Downsampling refers to the process of reducing the sampling rate of a signal, thereby representing the original signal with fewer data points. Its primary objective is to decrease the data volume and computational load while retaining sufficient information for subsequent analysis.

#### Artifact removal

EEG signals are inherently weak and highly susceptible to external interference, rendering the preprocessing stage particularly critical. The noise that disrupts standard EEG signals is collectively referred to as EEG artifacts, which can be primarily categorized into physiological and non-physiological artifacts [29]. Physiological artifacts result from voltage fluctuations caused by the subject’s head movements or activities in other adjacent areas (e.g., neck muscle activity, blinking, heartbeat). Non-physiological artifacts mainly originate from issues such as poor electrode-scalp contact, device-generated noise, or environmental interference (the environment around the device or the device inside the subject) [30]. The purpose of preprocessing is to maintain the integrity and authenticity of the data. While it is challenging to ensure completely artifact-free data, researchers have extensively developed methods to mitigate the effects of the artifacts [31]. Currently, several preprocessing approaches have been established.

##### Filtering

Digital filtering is a straightforward and efficient signal-processing technique that effectively extracts signals within specific frequency bands for targeted processing. It can be conceptualized as a frequency selection mechanism that allows signals of a specific frequency to pass through while preserving the desired frequency bands and attenuating signals in other frequency ranges. However, an inherent limitation of digital filtering is that ideal filter performance is challenging to achieve. In practical applications, while filters can remove artifacts, they may also inadvertently lead to the loss of some useful information [32].

##### Independent component analysis (ICA)

The ICA algorithm assumes that the signals are independent and aims to extract the signal of interest by decomposing the mixed signal into independent components. ICA can process non-Gaussian distributed signals without requiring the signals to satisfy the linear mixing assumption [33]. However, its limitations include the need for manual observation and identification of artifactual components for removal, which is both time-consuming and labor-intensive, potentially leading to inaccurate results. Additionally, the effectiveness of the algorithm is closely related to the amount of available data. Insufficient data may affect the decomposition.

##### Wavelet transform (WT)

WT is a powerful method that simultaneously analyzes the local characteristics of signals in both the time- and frequency-domains. It can accurately capture signal changes over time and frequency, extracting detailed information and approaching components, which aids in noise identification [34]. However, the effectiveness of WT is influenced by the choice of the wavelet basis function, with different functions being suitable for various types of signals. Additionally, factors such as its higher computational complexity must also be considered.

##### Canonical correlation analysis (CCA)

CCA is a statistical approach widely employed in EEG signal processing for artifact removal, particularly in mitigating electromyographic (EMG) interference [35]. By maximizing the correlation between multivariate signal sets, CCA enables the separation of artifact components from underlying neural activity [36]. Its primary advantage lies in its capacity to effectively address the non-Gaussian nature of EMG artifacts while preserving the integrity of relevant EEG information, making it highly suitable for applications requiring high signal fidelity. However, the computational complexity of CCA and its susceptibility to overfitting present significant challenges.

##### Multivariate empirical mode decomposition (MEMD)

MEMD, an extension of the traditional empirical mode decomposition (EMD), is specifically designed for multichannel signal analysis, rendering it highly effective for EEG artifact removal [37]. By decomposing signals into intrinsic mode functions (IMFs), while maintaining inter-channel coherence, MEMD is particularly well-suited to handle the non-linear and non-stationary characteristics of EEG signals [38]. Additionally, its independence from prior assumptions regarding artifact morphology enhances its versatility across diverse artifact scenarios. Nonetheless, the method faces notable limitations, including the intricacies of parameter tuning, stringent requirements for signal smoothness, and the potential for mode mixing. These challenges necessitate the implementation of robust preprocessing techniques and meticulous adjustment of decomposition parameters to ensure optimal artifact separation without compromising signal integrity.

##### DL

DL has demonstrated significant advantages in EEG signal denoising, becoming a research hotspot in recent years [39]. By training on large-scale EEG datasets, DL models can automatically learn complex signal features and noise patterns, achieving high-precision denoising performance. Unlike traditional methods, DL supports an end-to-end training framework, enabling the direct extraction of denoised output signals from raw EEG data [40]. This approach dramatically simplifies the complexity of multi-stage signal processing workflows. In this field, researchers have proposed various DL models, including recurrent neural networks (RNNs) [41], convolutional neural networks (CNNs) [42], and transformer architectures [43]. However, these autoregressive models inevitably face the over-smoothing problem in EEG denoising tasks, similar to challenges observed in visual and audio domains [44]. Generative adversarial networks (GANs) have been introduced into the EEG denoising domain as an effective solution to address this issue. Through adversarial learning strategies, GANs enable more accurate prediction of denoised signals. The generator can capture high-frequency signal features, mitigating the over-smoothing effect. At the same time, the discriminator constrains the differences between the generated and accurate signals, thereby enhancing the realism of signal restoration [45]. Incorporating auxiliary loss functions into GANs has improved denoising accuracy to some extent. However, this inevitably comes at the cost of increased model training complexity. In recent years, diffusion probabilistic models have emerged as a promising generative approach in EEG signal denoising [46]. Their core concept is based on a multi-step stochastic process using a Markov chain, progressively learning the noise degradation mechanism and reconstructing high-quality signals during the reverse diffusion. Compared to GANs, denoising diffusion probabilistic models exhibit higher training stability, as their loss function relies solely on optimizing a single model, avoiding the complexities associated with the joint training of generators and discriminators in GANs. More importantly, a previous study has shown that diffusion models preserve high-frequency signal information and generate fine-grained details, which are critical for the high-fidelity restoration of EEG signals [47].

##### DL and non-DL techniques for EEG artifact removal

DL and non-DL techniques each possess distinct advantages in removing EEG artifacts. DL approaches are highly automated and excel at handling complex and non-linear noise, particularly when multiple artifacts coexist. These methods do not rely on manual feature selection and are well-suited for large-scale data analysis. However, they typically require extensive labeled datasets, consume significant computational resources, and often lack model interpretability. In contrast, non-DL techniques involve lower computational costs, are easier to implement and interpret, and are suitable for applications with limited resources or stringent real-time requirements. Nonetheless, their performance is often limited when addressing complex or multi-source noise [48]. The advantages, disadvantages, and targeted artifact types of these methods are summarized in Table 2 [33–38, 41, 42, 45, 46]. Consequently, hybrid modeling strategies are gaining increasing attention. For example, Zeng et al. [49] proposed an EEMD-ICA method that first employs ensemble empirical mode decomposition (EEMD) to decompose potentially noisy multivariate neural data into IMFs. ICA is then applied to the IMFs to separate artifact components. Experimental results demonstrated that this method consistently outperformed comparable approaches regarding normalized mean square error and structural similarity. Additionally, Chen et al. [50] introduced a method combining EEMD with CCA for removing muscle artifacts from EEG signals. This approach effectively leverages inter-channel information. The authors tested it on a subset of randomly selected channels from multi-channel EEG data and achieved competitive results. Moreover, Gao et al. [51] proposed a dual-scale CNN-long short-term memory network (LSTM) model for artifact removal, which consists of three stages. First, a dual-branch CNN learns morphological features using convolution kernels of two scales. Second, the dual-scale features are enhanced by LSTM, which captures temporal dependencies. Finally, the extracted feature vectors are aggregated and passed through a fully connected layer to reconstruct artifact-free EEG signals. Experimental results indicate that this model has significant potential to achieve high-quality removal of unknown and mixed artifacts. Furthermore, Yin et al. [52] conducted a GAN-guided parallel CNN and transformer network (GCTNet). The generator integrates parallel CNN and transformer blocks to capture local and global temporal dependencies. The discriminator is then employed to detect and correct overall inconsistencies between clean and denoised EEG signals. The proposed network was evaluated on both semi-simulated and real datasets. Extensive experimental results demonstrated that GCTNet significantly outperforms state-of-the-art networks in various artifact removal tasks.

**Table 2:** Comparison of artifact removal algorithms

Methods	Type of artifact removal	Advantages	Disadvantages	References
ICA	EOG, EMG, ECG	Robust performance; No need for reference channels	Not applicable to single-channel EEG signals; Requires manual inspection	[33]
WT	EOG, EMG, ECG	Simultaneously displays both time-domain and frequency-domain information of the signal	Different parameter choices can lead to varying performance	[34]
CCA	EMG	Effectively handles the non-Gaussian characteristics of EMG artifacts; Retains more useful information while removing EMG artifacts	Computational complexity; Risk of overfitting	[35, 36]
MEMD	EMG	Capable of handling nonlinear EEG signals; Does not require prior knowledge of artifacts	Parameter selection is challenging and requires high smoothness of the signal	[37, 38]
CNN/RNN	EOG, EMG, ECG	End-to-end network; Achieving better performance than traditional algorithms	Many training parameters; Leading to over-smoothing issues	[41, 42]
GAN	EOG, EMG, ECG	The generator captures high-frequency signal features and suppresses excessive smoothing; The discriminator constraint enhances the authenticity of signal restoration	Joint training is complex; Sensitive to hyperparameters	[45]
Diffusion model	EOG, EMG, ECG	Stable model training; Exceptional fine-grained signal generation capability	The generation process exhibits high randomness; Requiring fusion and adjustment	[46]

*ICA* independent component analysis, *WT* wavelet transform, *CCA* canonical correlation analysis, *MEMD* multivariate empirical mode decomposition, *CNN* convolutional neural network, *RNN* recurrent neural network, *GAN* generative adversarial network, *ECG* electrocardiogram, *EMG* electromyographic, *EOG* electrooculogram, *EEG* electroencephalogram

#### Feature scaling

Feature scaling is essential to ensure that the dataset characteristics exhibit symmetric behavior, and one of the most commonly used methods is normalization. Certain machine learning algorithms may perform suboptimally with their objective functions when the range of raw data values fluctuates widely, and normalization can effectively address this problem.

### Feature extraction

Feature extraction is a crucial step in EEG signal analysis, as its quality directly impacts classification accuracy. In the era of big data, particularly within the medical domain, the primary objective of feature extraction is to achieve dimensionality reduction and data compression [53]. This methodology enables a more efficient representation of data using a reduced subset of features, ultimately enhancing the efficacy of AI algorithms in classification and diagnostic applications [54]. This section will focus on the commonly used feature types in EEG signal analysis, including time–frequency, temporal, spectral, spatial features, and DL architectures.

#### Time-domain features

Time-domain features are derived from calculations on raw EEG signals or signals preprocessed in the time-domain [55]. Although time-domain feature extraction is a relatively basic technique, it effectively quantifies changes in the signal over time, which is particularly important for EEG recordings that span several hours. Extracting time-domain features from specific time segments can significantly reduce the dimensionality of EEG signals. Below are some standard time-domain features, including statistical features [53] (such as mean, variance, mode, median, skewness, and kurtosis), entropy (such as Shannon entropy [56], approximate entropy [57], sample entropy [58], permutation entropy [59], and singular value decomposition entropy [60]), energy [61], power [62], fractal dimension [63], detrended fluctuation analysis [64], bandwidths [65] (amplitude modulation bandwidth and frequency modulation bandwidth), Hjorth parameters [66] (activity, mobility, and complexity), Hurst exponent [67], amplitude-integrated EEG [68], and zero-crossing rate [69].

#### Frequency-domain features

Frequency-domain analysis techniques offer a novel perspective by transforming signals from the time-domain into the frequency-domain, enabling a detailed understanding of the energy distribution across different frequencies. Additionally, frequency-domain features are often more stable than temporal features and can effectively reduce the impact of noise. Commonly used frequency-domain features include the Fourier transform [70], power spectral density (PSD) [71], and various metrics derived from different frequency bands (such as power, amplitude, and entropy [72]).

#### Time–frequency-domain features

Time–frequency-domain analysis methods integrate information from both the time- and frequency-domains, enabling localized analysis in the time–frequency-domain. Relying solely on either frequency- or time-domain feature is often insufficient, as these features are independent and cannot fully capture the characteristics of the signal. However, there is an inherent trade-off between time and frequency resolution, and researchers have made significant efforts to extract appropriate time–frequency features. Prominent methods include the short-time Fourier transform [73], WTs [74], and Hilbert-Huang transform [75].

#### Spatial-domain features

Spatial-domain feature extraction is a widely adopted and effective classification technique in EEG signal analysis, with one of the most well-known methods being common spatial pattern (CSP). CSP is a spatial filtering technique that transforms EEG signals into a unique spatial representation. In this representation, the variance within the same data group is minimized, while the variance between different groups is maximized due to potential inconsistencies in optimal frequency bands across subjects. However, CSP may not achieve ideal performance in all instances. Therefore, researchers have continuously developed variants of CSP to address these limitations, including filter bank CSP [76], correlation-based CSP [77], regularized CSP [78], filter band component regularized CSP [79], and time–frequency CSP [80], among others [81].

#### DL architectures

DL has emerged as a transformative technology in BCI research. DL enables end-to-end architecture design by integrating preprocessing, feature extraction, and classification tasks into a unified framework. This capability significantly reduces reliance on manual feature engineering and domain-specific expertise, paving the way for developing more robust and scalable BCI systems. Moreover, several widely recognized models have demonstrated their versatility [82–91]. However, due to the diversity of BCI tasks, different tasks often impose varying performance requirements on the models. This task-specific variability underscores the necessity of tailoring DL frameworks to meet specific objectives. Based on a previous study [54], we categorize EEG signal processing methods leveraging DL into 4 primary types: discriminative, representative, generative, and hybrid approaches.

##### Discriminative models

Discriminative DL models refer to architectures that learn features from input signals through non-linear transformations and classify them into predefined categories using probabilistic predictions. These models can perform feature extraction and classification simultaneously, making them widely applicable in EEG signal processing. Common examples of discriminative models include CNNs [82], RNNs [83], multilayer perceptrons (MLPs), and transformers [84].

##### Representative models

Representative DL models are architectures that focus on unsupervised feature extraction. These models are designed to uncover latent structures and features within the data, making them suitable for various tasks such as clustering and classification. Standard representative models include deep autoencoders [85], deep restricted Boltzmann machines [86], and deep belief networks [87].

##### Generative models

Generative DL models are typically used to generate, augment, or enhance training data by learning the underlying distribution of the data. These models can produce new samples to expand datasets or improve data quality, offering significant advantages in addressing issues such as data scarcity or imbalance. They have been widely applied in data augmentation, feature generation, and noise suppression. The most representative generative models include GANs and variational autoencoders [88].

##### Hybrid models

Hybrid DL models integrate two or more DL architectures into a single network to leverage the strengths of different models, thereby enhancing overall performance. Beyond the standalone DL models mentioned earlier, researchers have explored various approaches to combining DL networks. For instance, adding new modules [89], redesigning network structures [90], or modifying existing components [91] are common strategies to optimize model performance and address specific task requirements.

## Recent applications of EEG-based BCI in the medical field

**Fig. 1:**
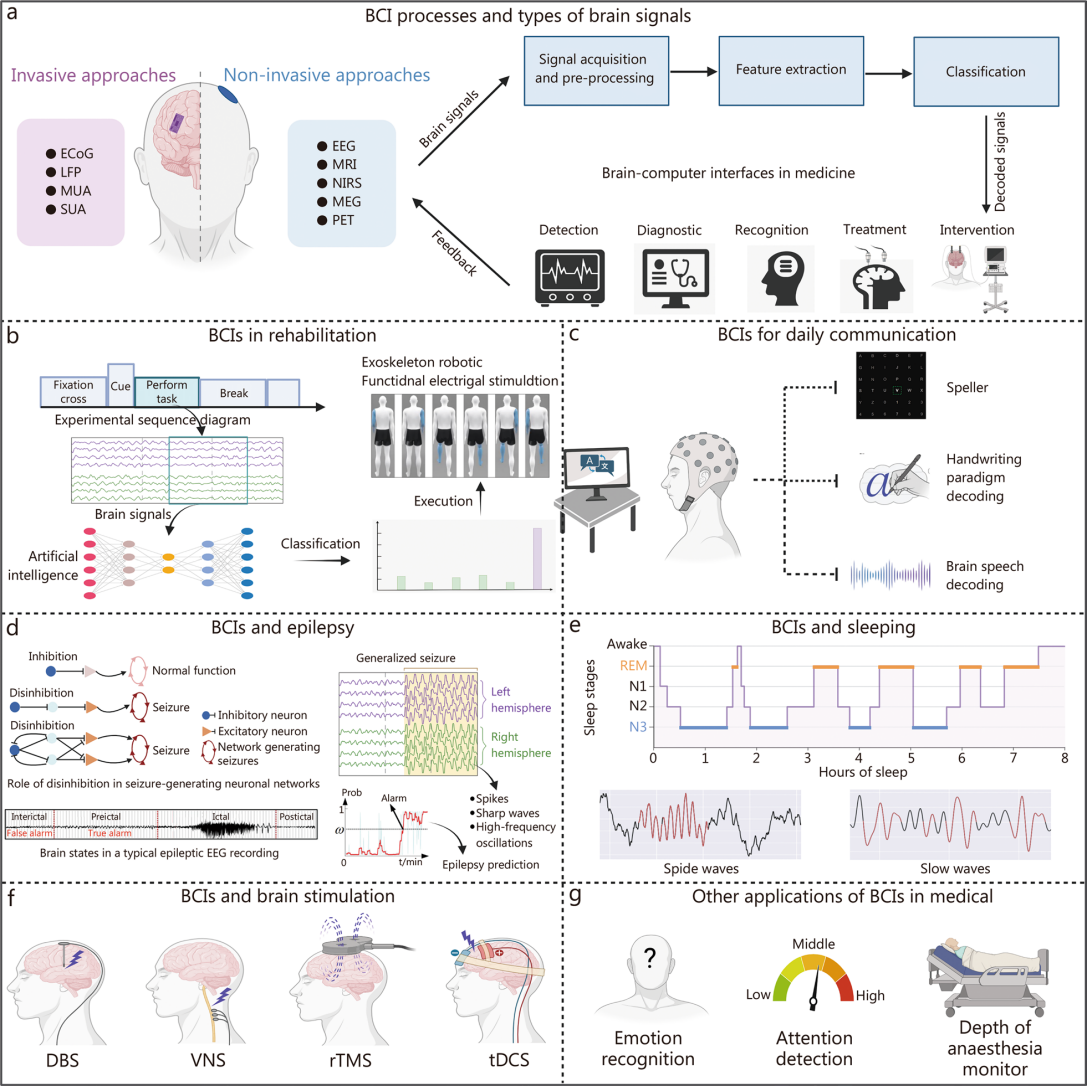
Applications of BCIs in healthcare. **a** The whole BCI process and the types of brain signals. **b** The process of BCI applications in rehabilitation, part of the figure by Ma et al. [92]. **c** The classification of BCIs based on the daily communication, part of the figure by Willet et al. [93]. **d** The role of inhibitory neurons in epilepsy, recorded EEG signals in seizure states, epilepsy prediction, and physiological markers of epilepsy, part of the figure by Daoud et al. [94] and Guo et al. [95]. **e** The sleep stages and the typical waveforms during sleep. **f** Four types of electrical stimulation of the brain for brain resuscitation and neurodegenerative diseases. **g** Other applications of BCIs in the medical field. ECoG electrocorticography, LFP local field potential, MUA multi-unit activity, SUA single-unit activity, EEG electroencephalogram, MRI magnetic resonance imaging, NIRS near-infrared spectroscopy, MEG magnetoencephalography, PET positron emission computed tomography, DBS deep brain stimulation, VNS vagus nerve stimulator, rTMS rhythmic transcranial magnetic stimulation, tDCS transcranial direct current stimulation, BCI brain-computer interfaces, REM rapid eye movement

This paper provides a comprehensive review of the applications of BCIs in the medical field. Figure 1 illustrates the overall process of BCIs, including rehabilitation applications [92], 3 daily communication methods [93], epilepsy monitoring and prediction [94, 95], sleep monitoring, 4 brain stimulation techniques for treatment, and other related applications. These sections offer a brief overview of the eight areas discussed in the paper. Table 3 also summarizes the features and limitations of these different areas.

**Table 3:** Comparison of different brain-computer interfaces in medical applications

Research field	Subdivided topics	Functions	Limitations
Rehabilitation	Rehabilitation—exoskeleton robotic	Enhanced mobility;	High cost;
		Promotes neuroplasticity;	Limited applicability;
		Personalized training	Technological limitations
	Rehabilitation—functional electrical stimulation	Promotes neuroplasticity;	Technical complexity;
		Improved motor function	Risk of skin irritation or infection;
			Cost and accessibility
	Rehabilitation—virtual reality	Enhanced patient engagement;	Not suitable for all patients;
		Multisensory stimulation;	Technical complexity and adaptation;
		Personalized and customized therapy	Cost and equipment challenges
Communication	Communication—speller	Generally non-intrusive;	Accuracy issues;
		Real-time communication;	Fatigue
		Ease of integration	
	Communication—the handwriting paradigm	Potential for faster input;	Complexity of signal interpretation;
		Customizable;	High cognitive load;
		Natural communication	Noise and signal interference
	Communication—the speech decoding	Natural communication;	Low accuracy and decoding challenges;
		High information transfer rate;	Training and calibration
		Integration with assistive devices	
Epilepsy	Seizure detection	Real-time alerts;	False positives/negatives;
		Early detection	Comfort and user compliance;
			Privacy and security concerns
	Seizure prediction	Early intervention;	Accuracy issues;
		Real-time monitoring;	Privacy and security concerns;
		Reduced risk of injury	Technological and sensor limitations
	Open-loop stimulation	Technologically mature;	Cannot adjust based on EEG activity;
		Does not rely on real-time monitoring	May lead to side effects
	Closed-loop systems	Real-time feedback and automatic adjustment;	Requires high-precision EEG monitoring;
		Reduces side effects;	Complex technology;
		Highly adaptive	Equipment cost and maintenance
Cerebral resuscitation	Diagnosis and evaluation	Enhanced clinical decision-making;	Difficult signal analysis;
		Assessment of brain function recovery;	Complex technology and equipment requirements
		Monitoring dynamic changes in brain function	
	Enhancing functional recovery	Enhanced mobility;	High cost
		Real-time communication	
Sleeping	Sleep detection	Real-time monitoring and feedback;	Poor tolerance for long-term wear
		Large and traceable data	
	Open-loop systems	Technologically mature;	Cannot adjust based on EEG activity;
		Does not rely on real-time monitoring	May lead to side effects
	Closed-loop systems	Real-time feedback and automatic adjustment;	Requires high-precision EEG monitoring;
		Reduces side effects;	Complex technology;
		Highly adaptive	Equipment cost and maintenance
Alzheimer’s disease	Diagnosis	Early diagnosis;	Complexity of data interpretation and analysis;
		Non-invasive	Patient adaptability issues
	Treatment	Early detection and intervention;	Technical complexity and high cost;
		Neurofeedback training	Dependence on equipment and technical support
Parkinson’s disease	Diagnosis	Early diagnosis;	Complexity of data interpretation and analysis;
		Non-invasive	Patient adaptability issues
	Treatment	Early detection and intervention;	Technical complexity and high cost;
		Neurofeedback training	Dependence on equipment and technical support
Anesthesia	Anesthesia depth monitoring	Real-time monitoring of anesthetic depth;	Interpretation and stability of EEG signals;
		Reduce drug usage and side effects;	Patient adaptability issues
		Enhance patient safety	
Others	Emotion recognition	Psychological health monitoring and intervention;	Accuracy;
		Safety monitoring and situational assessment	Privacy concerns;
			Simplification and misunderstanding of emotions
	Cognitive load assessment	Improve work efficiency and safety;	Limitations of evaluation methods;
		Personalized adjustment	Affected by environment and tasks;
			Individual differences
	Attention detection	Real-time monitoring;	Dependence on training;
		Wide application;	Signal noise and interference
		Personalized feedback	

### BCIs in rehabilitation

SCI, stroke, or other neurological conditions that result in loss of sensory and motor function significantly influence patients’ quality of life, often rendering them dependent on home care services for the rest of their lives [96]. Motor recovery is critical for alleviating the psychological and social challenges faced by these patients. BCI technology offers a direct communication pathway between the brain and external devices, which enables the decoding of neural activities and the translation of a patient’s intentions into actual motor control [97]. BCI-based rehabilitation systems provide patients with an innovative and efficient pathway for recovery. In this section, we will discuss the applications of BCIs in rehabilitation, specifically including the control of exoskeletal robots, functional electrical stimulation (FES), and virtual reality (VR) technologies, as illustrated in Table 4 [92, 98–114].

**Table 4:** Comparison of different brain-computer interfaces in medical applications

Research field	Subject	Method	Effect evaluation	References
Rehabilitation—exoskeleton—upper limb	14 patients with stroke in the subacute phase (Age: 25 – 75 years, 12 males)	The BCI-mediated exoskeleton training was performed 3 times per week for 4 weeks, while the control group only underwent conventional rehabilitation therapy	Patients in the BCI group recovered upper limb motor function better than the control group	[98]
	30 patients with stroke (Age: 18 – 80 years, 23 males)	4 weeks of BCI-based hand grasping/open motion training	Function-oriented and portable BCI training promotes hand rehabilitation after stroke	[99]
	22 healthy subjects [Age: (22.36 ± 3.53) years; 7 males]	10 different frequencies of non-dominant hand function training were carried out	The training duration of 30 min once a day for 5 d is probably the least effective dose to evoke cortical activation	[100]
Rehabilitation—exoskeleton—lower limb	7 subjects without motor disabilities	Research on walking control of assisted exoskeleton based on MI	EEG signals in the 14 – 19 Hz band provide higher accuracy	[101]
	12 healthy subjects [Age: (24.2 ± 1.5) years, 10 males]	EEG signals were recorded from subjects in normal and VR environments to verify whether VR could enhance lower limb active motor intentions in individuals with motor disabilities	VR enhances the detection of lower limb mobility intentions	[102]
	4 able-bodied subjects	A BCI system based on two paradigms of gamma-band activity and attention level in an MI mental task is proposed	Allows higher precision manipulation of exoskeleton robots for lower limb rehabilitation	[103]
	16 healthy and BCI-naïve human subjects [Age: (23.8 ± 1.9) years, 7 males]	Design of a BCI paradigm involving both unilateral lower limb stepping movements and contralateral upper limb swinging movements	Decoding step intent from composite limb tasks is feasible and superior to the traditional single lower limb paradigm	[92]
	6 healthy subjects (Age: 22 – 35 years, 2 males)	A cross-subject optimal selection algorithm for EEG channels is proposed	The algorithm selectively retains key channels and narrows the computational scope, thus significantly reducing computational resources	[104]
Rehabilitation—FES—upper limb	8 people with sub-acute SCI [Age: (55.4 ± 15.6) years, 6 males]	Constructed an arm therapy system combining EEG and FES for restoring hand function in tetraplegic patients	The BCI-FES has the potential to be used as a home hand therapy by people with SCI or stroke, as long as it is easy to use and provides support	[105]
	16 stroke patients in the sequela stage (Age: 22 – 72 years, 12 males)	FES system based on MI	Patients in the BCI group achieved greater functional improvement than those in the control group	[106]
	27 stroke patients (Age: 36 – 76 years, 16 males)	Comparing the efficacy of a BCI-based FES system with an FES system alone in stroke rehabilitation	The BCI-FES group showed a significant increase in upper limb motor function scores after treatment	[107]
	A patient with traumatic spinal cord injury resulting in quadriplegia (Age: 27 years, male)	An array was implanted in the patient’s left motor cortex, and machine learning techniques controlled the FES to stimulate the patient's forearm muscles so that they could perform upper limb movements such as grasping, pushing, and pulling objects	Using the BCI-FES, participants performed proficient and coordinated grasping and made clinically significant progress on upper extremity function tests	[108]
Rehabilitation—FES—lower limb	3 chronic strokes (Age: 59 – 83 years)	The BCI-FES was used to stimulate the proximal deep peroneal nerve in the paralyzed leg, and adjusted the stimulation parameters to achieve dorsiflexion of about 15°	Subjects all showed improvement in dorsiflexion after treatment	[109]
	25 individuals with chronic hemiparetic stroke [Age: (54.1 ± 14.7) years, 17 males]	Ankle dorsiflexion training of the anterior tibialis muscle in chronic hemiplegic stroke patients using the EEG-FES system	The results showed significant improvements in gait speed, step frequency, and step length on the less affected side with BCI-FES training	[110]
Rehabilitation—VR	5 healthy subjects (Age: 20 – 25 years, 3 males)	Development of a VR system combined with MI BCI specifically for upper limb rehabilitation in stroke patients	Healthy subjects were able to perform effective MI training with the system	[111]
	2 healthy right-handed participants	Proposed an MI-BCI system combining lower limb exoskeleton robotics and VR	Visual feedback provided by VR enhances lower limb rehabilitation training and facilitates neurological rehabilitation in brain-injured patients	[112]
	1 normal male subject	A real-time rehabilitation system for stroke patients was developed. The system integrates a VR game controlled by the BCI system	Feature extraction and classification schemes to achieve high accuracy and real-time response	[113]
	2 subjects	A real-time control system based on motion imagery is proposed. Using a low-cost BCI to control a robotic arm in VR	Controlling a robotic arm in VR by using a low-cost BCI	[114]

*BCI* brain-computer interfac, *EEG* electroencephalogram, *FES* functional electrical stimulation, *MI* motor imagery, *SSVEP* steady state visual evoked potentials, *VR* virtual reality

#### BCI-based exoskeleton robotic system

For patients with neurological disorders and sensory and motor loss, exoskeleton robots can be programmed to perform specific actions to help patients achieve their goals. BCI-mediated exoskeleton robots can also guide the patient’s paralyzed limbs to perform desired activities through feedback from brain signals, potentially stimulating damaged neural networks and promoting connectivity between different regions [115]. Thus, the BCI-based exoskeleton robotic system allows patients to actively control limb movement according to their intentions, rather than passively following pre-set motions. This approach can significantly enhance patients’ motivation to participate in training and improve rehabilitation outcomes [116].

##### Upper limb rehabilitation robots

The application of upper limb rehabilitation robots can be traced back to the 1990s [117]. With the advancement of BCIs, the efficacy and advantages of active upper limb rehabilitation robots are gradually being validated. For instance, Bhagat et al. [118] recruited 10 chronic stroke patients with stable baseline clinical scores to participate in a 12-session BCI-based robotic exoskeleton elbow training program. Each participant performed an average of (132 ± 22) repetitions per session. Post-training clinical assessments indicated significant improvements from baseline in the Fugl-Meyer assessment of the upper extremity and action research arm test scores, which increased by (3.92 ± 3.73) and (5.35 ± 4.62) points, respectively. Additionally, kinematic measurements demonstrated that participants’ movements became faster and more fluid. Next, Cantillo-Negrete et al. [119] recruited 7 subacute patients and 3 chronic stroke patients with severe upper limb impairments, who were randomly assigned to either 1 month of BCI therapy or conventional therapy. After BCI therapy, the scores for Fugl-Meyer assessment of the upper extremity and action research arm test were (23.1 ± 16) and (8.4 ± 10) points, respectively, while the scores after conventional therapy were (21.9 ± 15) and (8.7 ± 11) points, both significantly higher than the baseline scores of (17.5 ± 15) and (4.3 ± 6) points. Following this, Jochumsen et al. [120] recruited 11 healthy volunteers to participate in wrist extension activities using a motor imagery (MI) paradigm. Motor-evoked potentials (MEPs) elicited through transcranial magnetic stimulation were assessed before, immediately after, and 30 min following BCI training with the exoskeleton. Compared to pre-training measurements, the MEPs increased by (35 ± 60)% immediately after training and by (67 ± 60)% 30 min post-BCI training. Furthermore, Chen et al. [98] demonstrated the efficiency of BCI-mediated exoskeleton robotics by comparing stroke patients using this technology for rehabilitation (experimental group) with those receiving only conventional treatment (control group). The experimental group performed better recovery of upper limb motor function compared to the control group. Subsequently, another study conducted a 4-week BCI-based hand grasping/opening movement training program for stroke patients, indicating that functionally oriented portable BCI training facilitates hand rehabilitation post-stroke [99]. Finally, Lin et al. [100] conducted a randomized clinical trial with 16 young, healthy participants, who were divided into a high-frequency group and a low-frequency group. The high-frequency group underwent MI-BCI training once daily, while the low-frequency group trained every other day. All participants completed a total of 10 sessions of MI-BCI training. The results revealed that the high-frequency group exhibited stronger cortical activation and better BCI performance than the low-frequency group. Furthermore, compared to the low-frequency group, the high-frequency group showed increased cortical activation after 5 sessions of BCI training, with a significant enhancement observed after 10 sessions. In contrast, no similar effects were noted in the low-frequency group.

Numerous studies have demonstrated the scientific validity and practicality of BCI-based upper limb rehabilitation. However, designing a comfortable and efficient rehabilitation system remains a significant challenge for researchers. Lakshminarayanan et al. [121] developed a deployable BCI for controlling a virtual iTbot via a tablet. The experiment utilized EEG signals captured with a gel-free cap and processed through multiple stages, including signal validation, training, and testing. A real-time feedback system and virtual gaming environment were introduced during the testing phase, allowing participants to control the virtual iTbot using their EEG signals. This study highlights the potential of MI-based BCIs in robotic rehabilitation, particularly in enhancing engagement and personalization. Additionally, Bi et al. [122] proposed a migration data learning network to achieve cross-subject intention recognition for multi-class upper limb MI. Results from two public datasets showed that this model achieved state-of-the-art experimental performance. Moreover, Zhang et al. [123] constructed a user-friendly and comfortable hybrid BCI paradigm combining MI and high-frequency steady-state visual evoked potentials (SSVEP). They integrated a soft robotic glove as feedback to build a comprehensive “peripheral-central-peripheral” hand rehabilitation system.

Upper limb robots based on BCIs significantly improve functional recovery in patients. Researchers have also made considerable efforts to optimize AI algorithms and human–computer interaction. These advancements not only enhance rehabilitation outcomes but also lay a solid foundation for the future development of related research and applications.

##### Lower limb rehabilitation robots

Lower limb-powered robotic systems, such as exoskeletons and orthotics, have served as devices to assist or rehabilitate patients with walking disorders. These devices are typically controlled by non-natural specific physical actions, such as pressing a button or moving the body [124]. For example, robot-assisted gait training aims to regulate and correct real-time gait in hemiplegic patients by activating muscle coordination and neuroplasticity through repetitive motor coordination training [125]. BCIs enable the interaction between CNS and its external or internal environment by measuring CNS activity and converting it into artificial outputs to replace, restore, augment, supplement, or improve natural CNS outputs [126]. Thus, the usability and clinical relevance of these robotic devices can be further enhanced by BCI technology. To better integrate BCIs with exoskeleton robots, researchers have conducted extensive research. For example, Ferrero et al. [101] conducted a study on MI-based exoskeleton-assisted walking in 7 subjects without motor impairments. They found that EEG signals in the 14 − 19 Hz frequency band provided higher accuracy. Based on this finding, Dong et al. [98] verified that VR technology can enhance the detectability of lower limb movement intentions through a study of 12 healthy subjects. Additionally, Ortiz et al. [102] proposed a BCI system based on MI and user attention, allowing for more precise operation of an exoskeleton robot for lower limb rehabilitation. Ma et al. [92] also designed a composite limb BCI paradigm involving unilateral lower limb stepping actions and contralateral upper limb swinging actions to decode lower limb movement intentions. It was shown that decoding step intent from the composite limb task is feasible and superior to the traditional single lower limb paradigm.

The aforementioned studies have developed advanced strategies at both the paradigm and signal levels. Next, we will discuss the latest advancements in EEG signal decoding. Zhang et al. [127] proposed an integrated approach for classifying lower limb MI. This model combines various techniques to enhance classification performance, including multi-head self-attention and temporal convolutional networks. Additionally, a study conducted by Lu et al. [128] investigated continuous control decoding of rehabilitation robots based on a self-learning strategy. Utilizing a multimodal BCI paradigm, they developed an online classification model that decodes EEG signals into control commands, enabling continuous control of lower limb rehabilitation robots. Experimental results indicate that this method effectively reduces computational complexity and model redundancy, achieving an average recognition rate improvement of 17.82% per group. Subsequently, Lin et al. [129] designed a multi-state fusion neural network incorporating both closed- and open-eye states to predict BCI rehabilitation training outcomes for stroke patients after motor recovery. The study demonstrates that the predictive accuracy of the multi-state fusion network reaches 82%, representing a significant improvement over the unimodal model. Moreover, Wei et al. [104] proposed a cross-subject optimal EEG channel selection algorithm based on brain physiological functions. This algorithm selectively retains crucial channels and narrows the computational scope, significantly reducing computational resources. The results showed significant differences in θ and μ band energy of frontal channels between left- and right-side-driven MI tasks, providing new evidence that frontal regions significantly influence lower limb motor MI tasks.

Overall, the integration of BCI technology with rehabilitation robots can significantly facilitate the recovery of neural pathways and enhance patients’ autonomy and quality of life. As technology advances, these rehabilitation systems will become increasingly innovative, comfortable, and efficient.

#### BCI-based FES

FES can evoke action potentials in the motor nerves of paralyzed muscles and activate the somatosensory cortex involved in the motor neural control loop, allowing functional movement of the paralyzed limb. This process helps to improve mobility in patients with neurological disorders characterized by sensory and motor loss [130]. The BCI-based FES not only promotes rehabilitation through somatosensory feedback by stimulating distal limb muscles but also accurately identifies motor intention to control the FES apparatus, thereby completing the intended stimulation. This approach promotes the restoration of limb motor function and hand-grasping ability [18].

In recent years, the BCI-FES-based rehabilitation system has attracted significant interest from researchers. Zulauf-Czaja et al. [105] constructed an arm motor function recovery system that combines EEG and FES to restore hand function in paralyzed patients. In this system, FES is activated when real-time alpha (α) band power decreases below a certain threshold. Similarly, another study used an MI-based BCI system to instruct stroke patients to perform imagery wrist movements [106]. When the BCI detected the imagery of specified hand movements, FES was activated to stimulate muscle contraction, leading to wrist flexion and extension [106]. Furthermore, Biasiucci et al. [107] compared the efficacy of a BCI-FES-based system with FES alone in stroke rehabilitation. The results indicated that the BCI-FES group showed a significant increase in upper limb motor function scores after treatment compared to the control group, showing that BCI-FES is more effective for motor recovery in chronic stroke patients. Conversely, invasive studies have also been conducted. Bockbrader et al. [108] implanted the microelectrode array (MEA) in the left motor cortex of a patient with quadriplegia due to traumatic SCI. The patient’s motor intention was decoded using machine learning. Then, hand animations are generated on a computer-controlled the FES to stimulate the patient’s forearm muscles, enabling upper limb movements such as grasping, pushing, and pulling objects. The authors reported that the MEA-based BCI-FES system significantly improved the patient’s flexibility and coordination in manipulating objects and performing complex movements.

Compared to upper limb applications, fewer studies have focused on the use of BCI-FES in lower limbs due to the significant noise encountered during walking and standing. McCrimmon et al. [109] utilized BCI-FES to stimulate the proximal deep peroneal nerve in the paralyzed leg, adjusting the stimulation parameters to achieve a dorsiflexion of approximately 15°. Similarly, Chung et al. [110] employed an EEG-FES system to train ankle dorsiflexion by stimulating the anterior tibialis muscle in chronic hemiplegic stroke patients. Their results showed a positive effect on gait.

The healthy brain and neuromuscular system enable individuals to perform activities of daily living naturally [131]. However, for those experiencing functional impairments due to injury or illness, even simple tasks can become exceedingly challenging or even impossible to accomplish. Fortunately, BCI applications based on FES can effectively stimulate muscles and nerves, accelerate rehabilitation, and promote neural reorganization. Thus, these applications not only advance the field of rehabilitation medicine but also offer new hope to many patients, assisting them in returning to everyday life.

#### BCI-based VR system

BCI technology enables direct communication between the human brain and external devices by recording and decoding brain signals. VR technology significantly enhances the human–computer interaction experience by providing an immersive interactive environment [132]. Combining BCIs with VR technology can provide patients with a vivid feedback environment, thereby improving the effectiveness and engagement of rehabilitation training [133]. With the continuous progress of these two technologies, neurological rehabilitation training based on BCI-VR systems is receiving increasing attention. For instance, Wang et al. [111] developed a VR system combined with MI-BCI specifically for upper limb rehabilitation training of stroke patients. The system integrates real-life scenarios into monotonous rehabilitation training, offering 6 training scenarios and 9 training movements. This approach makes the training more engaging and effective through visual, auditory, and tactile multisensory feedback. Similarly, Su et al. [112] proposed an MI-BCI system incorporating a lower limb exoskeleton robot and VR. This system enhances the effectiveness of lower limb rehabilitation training and promotes neurological recovery in brain-injured patients through visual feedback. Furthermore, Aamer et al. [113] developed a real-time rehabilitation system for stroke patients, integrating a VR game controlled by a BCI system. Their study used multiple preprocessing, feature extraction, and classification schemes to achieve high accuracy and real-time response. As a result, the system performed well in terms of patient engagement and providing practical, immediate feedback. Moreover, Xu et al. [114] conducted a real-time control system based on MI using a low-cost BCI to control a robotic arm in VR. The findings highlighted the potential of low-cost BCIs in neurorehabilitation.

BCIs and VR technologies represent significant advancements in clinical rehabilitation. Continued research and development in this field promise to provide patients with more effective and engaging rehabilitation methods, thereby helping them regain motor function and improve their quality of life.

### BCIs for daily communication

Neurological disorders such as stroke, atresia syndrome, SCI, cerebral palsy, and multiple sclerosis can cause damage to central motor neurons, resulting in a loss of muscle control ability as well as functional and cognitive deficits. These conditions may also lead to the loss of communication abilities, increasing frustration and depression, further contributing to social disengagement [134]. BCI-based communication applications offer an effective solution to help patients express their will and intentions. Currently, BCIs for daily communication mainly utilize three modalities: spelling systems, handwriting paradigms, and speech decoding (Table 5) [93, 135–156].

**Table 5:** BCIs for communication functions

Research field	Subject	Method	Effect evaluation	References
Communication—speller—MI	2 subjects	The first BCI speller system based on MI to enter text by changing the orientation of the device	Develop an effective synchronized BCI system that uses the minimum number of controls (2) to control 30 targets (26 letters + punctuation)	[135]
	3 subjects (Age: 24 – 26 years, males)	A BCI speller based on 4 control commands was designed using advanced EEG decoding and NLP techniques	Three subjects achieved spelling rates of 3, 2.7, and 2 characters/min, respectively	[136]
	6 healthy subjects (Age:19 – 26 years, 4 males)	Design of an Oct-o-Spell paradigm incorporating intelligent input methods	PTE mode is more efficient than non-PTE mode	[137]
Communication—speller—P300	10 healthy male subjects	Designed a paradigm that includes modifications to the 9-key text interface	Significantly reduces word entry time and makes word entry easier	[138]
	10 participants [Age: (28 ± 4.84) years, 5 males]	A model is proposed that combines the two distinguishing features of 3D animation and the use of column flashes only	All participants declared in the subjective test that the proposed paradigm was more user-friendly than the classical paradigm	[139]
	9 subjects (Age: 20 – 34 years, 4 males)	Designed the first truly gaze-independent visual BCI, the stimuli consisted of colors displayed in widescreen, which made gaze focus independent of BCI spelling	The speller was tested online. Using 5 repetitions of the stimulus, the mean online symbol selection accuracy was 88.4%, and the mean online spelling speed was 1.4 characters/min	[140]
	25 subjects	A multi-window discriminative regular pattern matching method is proposed	The algorithm won first place in the 2019 World Brain-Controlled Robotics Competition	[141]
	Dataset I: gigascience			
Dataset II: scientific data	Designed filter banks and typical correlation analysis combination methods	Successful reduction of the number of P300 experiments good recognition rates and shorter recognition times in character recognition	–	[142]
	BNCI Horizon 2020 Database		–	
P300 Akimpech Database	Introducing a data alignment method in transfer learning	Data from different subjects can be more evenly distributed and inter-subject variability is effectively reduced		[143]
Communication—speller—SSVEP	22 healthy subjects (Age: 23 – 28 years, 9 males)	Design of a 120-target BCI spelling system based on coded modulated visual evoked potentials	Has a high average message rate (265.74 bits/min), while the equipment is large, fast, and has a short training time	[145]
	10 subjects [Age: (30 ± 5) years]	Design of a spelling system incorporating EMG signals and SSVEP paradigms	Provide a user-friendly, practical system for speller applications	[146]
	20 healthy participants (Age: 24 – 46 years, 16 males)	Proposed design of a stimulus-responsive hybrid speller using eye tracking with EEG and video	The authors indicated that this set produced less fatigue, worry, strain, and discomfort	[147]
	12 healthy subjects (Age: 20 – 26 years, 5 males)	Improvements to the SSVEP typing system in three areas: the user interface, the EEG acquisition device, and the decoding algorithm	Make the typing system more flexible and more suitable for practical application scenarios	[148]
Communication—speller—hybrid	14 healthy subjects (Age: 18 – 41 years, 8 males)	A synchronous hybrid system based on P300 and SSVEP	The average online utility information transfer rate achieved using this method was 53.06 bits/min	[149]
	11 healthy volunteers (Age: 19 – 24 years, males)	A hybrid speller in the frequency-enhanced row-column paradigm is proposed, which allows the simultaneous triggering of two signals: the P300 and the SSVEP	The average accuracy of the proposed hybrid BCI is 94.29% and the information transfer rate is 28.64 bits/min	[150]
	20 subjects	A hybrid BCI-speller system is proposed, encoded by a combination of EEG functions: the P300, motor-visual evoked potentials, and SSVEP, and using a layout of more than 200 targets	The online prompted spelling and free spelling results show that the proposed hybrid BCI speller achieves average accuracies of (85.37 ± 7.49)% and (86.00 ± 5.98)% for the classification of 216 targets, with average message transfer rates of (302.83 ± 39.20) bits/min and (204.47 ± 37.56) bits/min, respectively	[144]
	54 subjects	A hybrid BCI based on a two-stream convolutional neural network is presented	In single mode (70.2% for MI and 93.0% for SSVEP) and mixed mode (95.6% for MI-SSVEP mixed mode)	[151]
Communication-the handwriting paradigm	5 healthy right-handed participants [Age: (30.83 ± 1.8) years, males]	Subjects were asked to write “Hello, world!” repeatedly on a tablet. Then machine-learning methods were used to recognize the EEG signals	The results suggest that the possibility of recognizing handwritten content directly from brain signals	[152]
	A participant with hand paralysis due to spinal cord injury	Demonstration of an intracortical BCI that attempts to decode handwritten actions from neural activity in the motor cortex and translate them into text in real time	The authors showed that the subjects’ typing speeds were comparable to the mobile phone typing speeds of smart people in the participants’ age group	[93]
	The handwriting BCI dataset was publicly released by the Stanford University research team of Willett et al. [93]	A temporal channel cascade transformation network is proposed to decode neural activity for imagining handwriting movements	In the imaginary single character recognition task, the recognition accuracy of the proposed model can reach 95.78%, which is 2% better than the existing state-of-the-art model	[153]
Communication—the speech decoding	A speech-impaired individual with ALS	A chronically implanted BCI was used to synthesize comprehensible words online	Results show that 80% of synthetic words can be correctly recognized by human listeners	[154]
	An ALS patient	Implantation of electrocorticography into the sensorimotor cortex allows patients to operate computer applications with 6 intuitive voice commands	The results show that speech commands can be accurately detected and decoded without the need to recalibrate and retrain the model	[155]
	48 participants (22 males)	Development of a novel differentiable speech synthesizer	The ability to process data with different spatial sampling densities and to simultaneously process EEG signals from the left and right brain hemispheres	[156]

*BCI* brain-computer interface, *EEG* electroencephalogram, *MI* motor imagery, *SSVEP* steady state visual evoked potentials, *ALS* amyotrophic lateral sclerosis, *EMG* electromyographic, *PTE* predictive text entry, *NLP* natural language processing

#### Speller-based BCI communication system

BCI spellers enable users to spell words by selecting letters on a computer screen. The paradigms of BCI spellers mainly fall into three categories: MI, P300, and SSVEP [157]. However, each paradigm has its limitations, which has prompted the development of hybrid BCI spellers that integrate the advantages of multiple approaches.

##### MI spelling system

MI-based BCIs provide a non-muscular communication system for individuals with disabilities, requiring no external stimulation. The first MI-based BCI, named Hex-o-Spell [135], was introduced in 2006. This system simulated the layout of a telephone keyboard to facilitate typing. Its goal was to develop an effective synchronized BCI system that uses a minimum number of controls (2) to select from 30 targets (26 letters + punctuation). Six hexagons are arranged around a circle, with an arrow pointing from the center to one of the hexagons. The 30 characters are evenly distributed within these hexagons, each containing 5 characters and 1 blank space. By imagining the movement of the right hand or foot, users could rotate the arrow or select the hexagon it points to. If an error occurs during the initial selection, the blank side of the hexagon provides an option to return to the first stage. Complete words are then spelled out [158]. This complex paradigm necessitates the development of intelligent user interfaces and natural language processing techniques for spelling prediction. For example, D’Albis et al. [136] conducted an innovative MI spelling system. The graphical user interface consists of 4 rectangles positioned at the edges of the screen. The top, left, and right boxes contain English characters. In contrast, the bottom box provides commands to assist users in controlling the speller, such as undo, delete, toggle between letters and numbers, and exit the interface. Users select the left and right boxes by imagining the movement of their left and right arms, respectively; the top box by imagining both arms moving simultaneously, and the bottom box by imagining leg movements. Furthermore, they incorporated natural language processing techniques to reduce the number of selection steps required. Similarly, Perdikis et al. [159] proposed an adaptive BCI speller based on MI that improves performance by leveraging application-derived context and adapting both the classifier and the spelling process. Furthermore, Cao et al. [137] developed an Oct-o-Spell paradigm combined with an intelligent input method, with an interface similar to Hex-o-Spell [135]. The authors indicate that the predictive text entry paradigm is more efficient than the non- predictive text entry paradigm.

##### P300 spelling system

The P300 spelling system features a screen with characters arranged in a matrix format, allowing users to select characters based on event-related potential (ERP) responses. This response manifests as a positive wave in brainwave activity approximately 300 ms after the selection. Farwell and Donchin [160] introduced this P300-based spelling system for character recognition in 1988. In the P300 paradigm, each row and column flashes sequentially to identify the target character. Participants must focus their attention on the desired letter. When the row and column containing the target symbol flash, it elicits a P300 wave in the EEG signal. Although typing speed using this method may be slower compared to that of healthy individuals, it is essential for those who cannot communicate through other means.

Over the years, technological advancements have enabled P300 spellers to evolve towards faster, more accurate, and more user-friendly designs. Classic P300 speller paradigms include the row-column paradigm [161], single character paradigm [162], region-based paradigm [163, 164], and chromatic speller [138]. For example, Obeidat et al. [165] introduced a novel P300 spelling method known as the edge paradigm (EP). Unlike existing P300 spelling methods, EP provided an adjacent square at the outer boundary of the matrix for each column or row, thereby reducing the effects of crowding and adjacency by substituting this square for each flash of a row or column. The results indicated that in copy-spelling mode, 14 neurologically typical participants achieved a higher accuracy of (93.3 ± 2.0)% using EP compared to (81.7 ± 2.8)% with the row-column paradigm. Furthermore, EP enhanced communication speed, and subjective evaluations showed that it significantly reduced fatigue while improving comfort. Similarly, Akram et al. [139] developed a P300 typing interface based on the “9-key text” (T9), similar to a mobile phone keyboard. Users enter initial characters through a 3 × 3 matrix interface, and the system suggests candidate words based on the entered characters. The user can then select a suggestion to complete word input. In an experiment involving 10 participants, the traditional spelling system required an average of 3.47 min to enter 10 random words, while the proposed system reduced this time to an average of only 1.67 min under the same conditions, demonstrating an efficiency improvement of 51.87%. Moreover, Korkmaz et al. [140] proposed a novel spelling paradigm that integrates two significant features: three-dimensional animation and the exclusive use of column flickering. Compared to the traditional two-dimensional paradigm based on row and column flickering, this new approach offers enhanced functionality. The experiments evaluated the effectiveness of detecting P300 waves and recognizing target characters using single or multiple combinations of EEG electrodes over 1, 3, and 15 flashes. The results showed that satisfactory performance could be achieved with fewer electrodes. Additionally, all participants rated the proposed paradigm as easier to use than the traditional one. Besides, Medina-Juliá et al. [166] noted that for individuals with severe motor impairments affecting head and eye movement, the size of the spelling device may pose limitations. To assess the usability of three different sizes of spelling interface, the authors recruited 7 patients with ALS and 1 with Duchenne muscular dystrophy. The results indicated that the medium-sized speller was significantly more practical in online testing and efficient in workload tests, achieving the highest satisfaction levels among users.

Additionally, researchers have focused on enhancing the speed and accuracy of EEG signal decoding. For instance, Xiao et al. [141] proposed multi-window discriminant regularized pattern matching, which won first place in the 2019 world BCI competition. Building on this, Blanco-Díaz et al. [142] utilized filter banks and CCA methods to reduce the number of P300 experiment repetitions, thereby achieving more accurate and faster recognition results. Furthermore, Liu et al. [143] introduced a data alignment method in transfer learning to evenly distribute data from different subjects, effectively reducing inter-subject variability. With advancements in technology and algorithms, P300 speller systems continue to be optimized, providing users with more efficient communication methods.

The P300 waveform is a significant discovery in EEG signal analysis with a wide range of applications. However, P300 spelling systems exhibit certain limitations. Firstly, all stimuli need to be executed before each output, resulting in reduced efficiency. Additionally, the amplitude of P300 potential decreases over time, reducing classification accuracy.

##### SSVEP spelling system

With advancements in EEG technology, research has demonstrated that when subjects fixate on repetitive stimuli presented at a constant frequency, SSVEP emerges in EEG signals. This has attracted considerable attention, and SSVEP-based spelling systems have seen significant improvements, including larger instruction sets, higher decoding accuracy, and faster response times [165]. Tianjin University has demonstrated exceptional performance in this field, breaking the 100-command set boundary for the first time [167] and further advancing to a 216-command set in 2023 [144]. Moreover, Sun et al. [145] designed a BCI spelling system based on coded visual evoked potentials with 120 targets, achieving a high average information rate of 265.74 bits/min. This system features a large-scale device, high speed, and short training time. In a related effort, Sadeghi et al. [146] developed a speller system that integrates EMG signals with the SSVEP paradigm to enhance the information transfer rate. Mannan et al. [147] proposed a hybrid speller utilizing EEG and eye-tracking technology to improve user comfort in scenarios with numerous flashing stimuli. Additionally, Tang et al. [148] enhanced the typing system by focusing on the user interface, EEG acquisition device, and decoding algorithm, making it more flexible and suitable for practical application scenarios.

##### Hybrid spelling system

Hybrid spelling systems are developed to exploit the strengths of two or more paradigms [168, 169]. For example, Yin et al. [149] reported a simultaneous hybrid system that identifies target items using two-dimensional coordinates based on P300 and SSVEP, achieving an average information transfer rate of 53.06 bits/min. Expanding on this, Bai et al. [150] proposed a hybrid speller in the frequency-enhanced rows and columns paradigm, which simultaneously elicits both P300 and SSVEP signals. The authors devised different blink frequencies for each row and column to identify the target stimulus once the classifier recognizes the selected columns and rows. This hybrid BCI achieved an average accuracy of 94.29% with an information transfer speed of 28.64 bits/min. In another development, Han et al. [144] from Tianjin University introduced a hybrid BCI-spelling system encoded by a combination of EEG functions: P300, motor-visual evoked potentials, and SSVEP, constructing a layout of over 200 targets. The authors noted that motor-visual evoked potentials and P300 components are significantly expressed in the central, temporal, and occipital regions, while SSVEP features are most distinctive in the occipital region. Online prompted spelling and free spelling results showed that the proposed hybrid BCI speller achieved average accuracies of (85.37 ± 7.49)% and (86.00 ± 5.98)%, respectively, for the classification of 216 targets, with average information transfer speeds of (302.83 ± 39.20) bits/min and (204.47 ± 37.56) bits/min, respectively. In addition to these hybrids triggering paradigms, Luo et al. [151] proposed a hybrid BCI based on a two-stream convolutional neural network (TSCNN), combining the SSVEP and MI paradigms. The TSCNN automatically learns to extract EEG features from both paradigms during training, improving decoding accuracy by 25.4% compared to the MI paradigm and by 2.6% compared to the SSVEP paradigm in the test data. Furthermore, TSCNN demonstrated comparable performance in both single-mode scenarios (70.2% for MI and 93.0% for SSVEP) and mixed-mode scenarios (95.6% for MI-SSVEP mixed-mode), verifying its generality.

Different paradigms of spelling systems in BCI technology each possess unique advantages. The MI spelling system does not require external visual stimuli, making it suitable for specific applications. The P300 system enhances signal clarity and accuracy through the use of single-frequency stimuli, while the SSVEP system typically achieves faster spelling speeds via varied frequency stimulation. Hybrid BCI systems integrate multiple paradigms to improve spelling flexibility and accuracy. However, each system has its limitations, which are detailed in Table 6.

**Table 6:** Advantages and disadvantages of different spelling paradigms

Spelling systems	Advantages	Disadvantages
MI	Without external stimulation;	Requires the users to undergo specialized training;
	Suitable for specific applications	Typing speed is relatively low;
		Prolonged use can cause fatigue
P300	Stimulating with a single frequency signal;	Reaction time is relatively long;
	Clear signals	Relies on external visual stimuli
SSVEP	Relies on different frequency stimuli;	When the frequency is close or harmonics are present, performance decreases;
	Enabling real-time typing;	May cause user fatigue
	Fast typing speed, large command set	
Hybrid	Combining the advantages of multiple BCI paradigms;	Increasing user cognitive load;
	Improving typing accuracy and flexibility	High system complexity increases the computational burden

*MI* motor imagery, *SSVEP* steady state visual evoked potentials

#### The handwriting paradigm-based BCI communication system

In recent years, the detection of handwriting trajectories through neural signals has garnered significant attention. This approach not only enhances the user experience but also opens up new avenues for biometric authentication by recognizing the unique neural signature associated with handwriting. Additionally, individuals with severe motor impairments could greatly benefit from this BCI technology, which can significantly improve their communication and interaction capabilities. Notably, Pei et al. [152] recruited 5 participants to repeatedly write “Hello world!” on a tablet while brain signals were recorded using an EEG device. Machine learning methods were then employed to recognize the written symbols. The experimental results indicated that the accuracy of the CNN-based classifier ranged from 76.8 to 97.0% for the same participant, and cross-subject accuracy ranged from 14.7 to 58.7%. These findings suggest the potential for recognizing handwritten content directly from brain signals. Additionally, Willett et al. [93] demonstrated an intracortical BCI that uses a novel RNN approach to decode handwritten actions from neural activity in the motor cortex and translate them into text in real-time. A participant with SCI, whose hands were paralyzed, achieved a typing speed of 90 characters/min with an online accuracy of 94.1%. The authors noted that this speed was comparable to the typing speed of a normal person of the same age using a mobile phone, which is approximately 115 characters/min. The Clinical Translational Research Center for brain-computer regulation at Zhejiang University conducted research on implantable BCIs for a paraplegic patient. This patient is now able to perform complex tasks such as drinking water, eating, and controlling a robotic arm to write Chinese characters through MI. Further improvements to decoding algorithms have also attracted considerable attention. Zhou et al. [153] proposed a temporal channel cascade transformer network to decode the neural activity of imagery handwriting motion, enabling recognition of imagery handwriting based on spiking activity recorded by MEAs. The authors evaluated the performance of their framework using both single-character and sentence-level handwriting imagery datasets from a public BCI dataset. The comparative results demonstrated the superiority of the proposed framework and strategy.

#### Speech decoding-based BCI communication system

Damage or degeneration of speech and other motor pathways, such as brainstem strokes or ALS, may interfere with effective communication without affecting the brain regions responsible for speech or cognition [170]. With recent advances in machine learning and neural recording techniques, BCIs offer a promising avenue to extend beyond cursor control and text generation on computers, enabling real-time speech synthesis. The potential of BCIs for speech synthesis has only recently been recognized, thanks to pioneering studies that utilized intracranial ECoG recordings from patients undergoing epilepsy surgery. These studies indicate that the cortical areas responsible for phonation and articulation are distributed across a large portion of the ventral sensorimotor cortex [154–156, 170–172]. Using sufficiently dense and comprehensive electrode arrays makes it possible to decode speech and reconstruct its sound from ECoG signals [171]. In a notable study, Angrick et al. [154] used a chronically implanted BCI to synthesize words online, demonstrating that human listeners could correctly recognize 80% of the synthesized words. This shows that individuals with ALS can use a BCI to generate words while retaining their vocal characteristics, providing further evidence of the stability of speech-based BCIs. In another study, Luo et al. [155] performed a 3-month clinical trial on an ALS patient. The researchers implanted ECoG electrodes into the sensorimotor cortex, enabling the patient to operate a computer application via 6 intuitive voice commands. Experimental results exhibited that these voice commands could be accurately detected and decoded without recalibrating or retraining the model. This demonstrates the accuracy and stability of the ECoG implant-based speech BCI system in controlling external devices. Furthermore, Chen et al. [156] developed a novel differentiable speech synthesizer that employs a lightweight CNN to encode speech into interpretable parameters such as pitch, loudness, and resonance peak frequency and resynthesizes the speech using the same synthesizer. By mapping neural signals to these parameters, the researchers constructed an interpretable and data-efficient neural speech decoding system. The system demonstrated high reproducibility among 48 participants, handling data with varying spatial sampling densities and simultaneously processing EEG signals from both left and right brain hemispheres, showcasing its strong potential in speech decoding. Card et al. [172] recently reported a study involving a 45-year-old patient with ALS. The patient underwent surgery to implant 4 MEAs. On the 25th day after surgery, the system was used, the research team collected and processed 30 min of cortical recordings while the participant attempted to speak. At this stage, the neural prosthesis achieved 99.6% accuracy with a vocabulary of 50 words. After 1.4 h of system training on the following day, the neural prosthesis achieved 90.2% accuracy with a vocabulary of 125,000 words. With further training over 8.4 months post-implantation and 248 cumulative hours of use, the neural prosthesis maintained an accuracy of 97.5% during self-paced conversations.

BCI technology offers new communication methods for individuals who have lost the ability to interact with others due to illness or other reasons. Among these, non-invasive brain-controlled spelling technology is widely used as a primary means of communication. Although handwriting paradigm decoding and speech decoding techniques are typically invasive, they demonstrate significant potential in enhancing communication capabilities. As technology advances, the application domains of BCIs are expanding. In the future, the integration of advanced technologies such as AI and DL will render these communication methods more precise and efficient, thereby further improving the quality of life for patients and helping them reconnect with society.

### BCIs in epilepsy diagnosis and treatment

Epilepsy is a chronic neurological disorder characterized by abnormal neuronal discharges in the brain [173]. Seizures associated with epilepsy impose significant psychological and physiological burdens on patients and their families, affecting approximately 50 million people worldwide [174]. The most common method for treating epilepsy is pharmacotherapy. However, this approach is ineffective for about one-third of patients [175]. In recent years, BCI technology has made significant progress in epilepsy diagnosis and treatment. This section will explore the detection and prediction of epileptic seizures using BCIs, as well as the application of open-loop and closed-loop neural modulation techniques in managing epilepsy.

#### Seizure detection

Epileptic seizure detection systems can automatically identify epileptic seizure events, thereby providing clinicians with critical information for treatment planning. These systems can also issue alerts to reduce the harmful effects of seizures on patients. BCI-based epilepsy seizure detection algorithm mainly involves two processes: epileptic EEG feature extraction and automatic seizure detection. The formal includes parameters such as frequency band power, brain area energy, and global synchronization levels [176, 177]. AI algorithms, including support vector machines [178], random forests [179], CNN [180], graph convolutional network (GCN) [181], or transformer [182], are employed to classify features related to epileptic seizures and determine the patient’s seizure status.

Evaluation metrics for epileptic seizure detection systems include sensitivity, false positive rate, detection latency, and computational complexity [183, 184]. Impressively, Burrello et al. [185] proposed Laelaps, an efficient and rapid learning algorithm for detecting seizures from long-term intracranial EEG signals. Laelaps employs symbolic dynamics and brain-inspired hyperdimensional computing to perform end-to-end binary operations. It successfully detected 116 epileptic seizures without false positives in the analysis of 2656 h of intracranial EEG recordings from 18 patients with drug-resistant epilepsy, achieving an average sensitivity of 85.5% and an average detection latency of 18.9 s. The Laelaps algorithm consumes between 32 to 35 milli-joules per classification on the Nvidia Tegra X2 embedded device, making it suitable for long-term implantable devices powered by batteries. After years of algorithmic development, Ahmad et al. [186] conducted a hybrid DL approach for detecting seizures in EEG signals. This method comprises two core components. First, it utilizes the K-means synthetic minority oversampling technique to balance the dataset. Second, it integrates a one-dimensional CNN with a bidirectional LSTM using truncated backpropagation through time, effectively extracting spatial and temporal sequence information while reducing computational complexity. The experimental results on the publicly available University of California Irvine seizure recognition dataset show that this algorithm outperforms several baseline DL algorithms and state-of-the-art techniques in terms of accuracy, sensitivity, specificity, and F1 score. Furthermore, Pan et al. [187] combined the EMD of EEG signals with their corresponding PSD, significantly enhancing the decoding performance of neural networks in scenarios with limited sample sizes. Specifically, they used the IMFs obtained from EMD decomposition along with their PSD as inputs for the neural network, while carefully designing the architecture of CNNs. Experimental results indicated that even when the number of training samples was reduced to 10%, this method still achieved satisfactory decoding performance, demonstrating its potential for application in resource-constrained settings.

#### Seizure prediction

Compared to seizure detection, predicting epileptic seizures may hold greater research value [188]. Studies have shown that identifiable features can be observed in EEG signals prior to an epileptic seizure, such as the appearance of spikes, sharp waves, and high-frequency oscillations (HFOs), which can serve as potential biomarkers [189, 190]. In clinical practice, interictal epileptiform discharges are not always detectable, and EEG recordings may appear normal even in the presence of an underlying epileptic disorder, leading to diagnostic challenges [191]. Therefore, identifying alternative biomarkers would be highly beneficial, as this would enable reliable predictions of epilepsy in individuals, even in the absence of overt epileptic activity. Through continuous exploration and validation by researchers, several candidate EEG biomarkers have been identified.

HFOs are transient oscillatory field potentials ranging from 80 to 500 Hz, first recorded over two decades ago in the hippocampus of rats and the human entorhinal cortex. HFOs can be categorized into physiological HFOs (80 − 250 Hz) and fast HFOs (250 − 500 Hz). Physiological HFOs typically occur during sleep [192] and are closely associated with normal cognitive functions such as memory processing [193]. Fast HFOs have been linked to epilepsy, and numerous studies in both animals and humans, primarily utilizing intracranial recordings, have been conducted over the years. These studies aim to better delineate the seizure onset zone and establish the clinical significance of fast HFOs [192–194].

The second aspect involves changes in brain connectivity states, typically measured through two approaches: functional connectivity and effective connectivity. Functional connectivity refers to the interactions between specific brain regions and is often used as an indicator for identifying potential diseases. Effective connectivity, on the other hand, clarifies how information is transmitted from one specific brain region (referred to as a driver) to another (referred to as a receiver). Coito et al. [195] conducted a comparative analysis of high-density EEG recordings from 20 patients with left temporal lobe epilepsy, 20 patients with right temporal lobe epilepsy, and 20 healthy controls. Their results indicated significant differences in connectivity metrics between patients and healthy subjects. In another study, Carboni et al. [196] examined 49 adult patients with focal epilepsy and 16 healthy controls using high-density EEG and structural MRI. Their findings revealed that even during interictal periods without epileptiform discharges, there were changes in connectivity within the entire brain and specific resting-state networks.

Therefore, early research efforts primarily focused on extracting features from the pre-ictal phase of a seizure, and an alert was triggered when feature values exceeded the predefined threshold [197]. Additionally, researchers classified EEG recordings into interictal and preictal periods. By analyzing EEG signals during these two phases, they determined whether the patient was in a preictal state, thus providing timely alerts [94]. In 2016, Brinkmann et al. [198] launched an open competition on the Kaggle platform using a crowdsourcing approach to classify continuous multi-day intracranial EEG recordings from humans and canines into pre-ictal or inter-ictal phases (in this study, at least 1 week away from the seizure event). The best-performing algorithm achieved an accuracy rate of 0.81. Building on this progress, Gao et al. [199] proposed a self-explanatory DL model for patient-specific seizure prediction: a multiscale prototype partial network. This model measures the similarity between inputs and prototypes as evidence for making final predictions, providing a transparent reasoning process and decision-making basis. The proposed model was evaluated on two public epileptic EEG datasets [Children’s Hospital of Boston-Massachusetts Institute of Technology (CHB-MIT) and Kaggle], achieving state-of-the-art performance with reliable evidence. Furthermore, Guo et al. [95] developed an innovative epileptic seizure prediction system called contrastive language-image pre-training. This system utilizes contrastive learning, spatiotemporal spectral networks, and dynamic GCNs to effectively extract features from EEG signals and dynamically capture the spatial relationships between electrodes, significantly improving the accuracy and cross-subject generalization capability of epileptic seizure prediction. On the CHB-MIT database, which includes EEG recordings from pediatric patients with refractory seizures collected at Boston Children’s Hospital, contrastive language-image pre-training achieved a sensitivity of 96.7% and a false positive rate of 0.072 per hour. The system’s sensitivity on the Xuanwu Hospital intracranial EEG database was 95%, with a false positive rate of 0.087 per hour.

In recent years, with the rapid advancement of modern medicine, approximately 70% of epilepsy patients can achieve a seizure-free life through appropriate diagnosis and treatment. However, 30% of patients still face drug-resistant epilepsy that cannot be controlled by medication [198]. Therefore, research on seizure prediction is of paramount importance, as it has the potential to significantly improve the quality of life for epilepsy patients. In the future, continuous advancements in neuroimaging, neurophysiology, and AI are expected to significantly enhance the accuracy and real-time capabilities of seizure prediction. Meanwhile, the development of wearable devices provides new possibilities for the daily monitoring of epilepsy patients, making the application of real-time prediction systems increasingly feasible.

#### Open-loop stimulation

Open-loop stimulation is a neuromodulation treatment method primarily used in epilepsy therapy through devices such as vagus nerve stimulator (VNS) or deep brain stimulation (DBS) [200]. These devices activate according to a preset protocol without involving direct feedback between the brain state and the stimulation protocol [201]. For VNS, the stimulator is typically implanted in the patient’s chest or neck via surgery, with electrodes wrapped around the vagus nerve. For DBS, electrodes are implanted in specific areas of the brain, such as the anterior thalamic nuclei (ATN), through stereotactic surgery. Depending on the patient’s specific condition, doctors need to program the stimulator, setting specific parameters for stimulation frequencies, intensities, durations, and cycles. In support of this, Grasl et al. [202] demonstrated that VNS implantation is an efficient and safe method for treating drug-resistant epilepsy. Their study showed that 76.7% of patients experienced at least a 50% reduction in seizure frequency, 72.1% exhibited improved clinical global impression scores, and 18.6 – 60.5% demonstrated significant improvements in various aspects of quality of life. Further advancing the understanding, Freund et al. [203] hypothesized that the proximity of DBS electrodes to the ATN-mammillothalamic tract (MMT) connection determines the response of drug-resistant epilepsy patients to ATN DBS treatment. The study found that responders (patients with at least a 50% reduction in seizure frequency) had electrodes closer to the ATN-MMT connection than non-responders. This research supports the hypothesis of directly targeting the ATN-MMT connection, emphasizes the importance of precise targeting and image-based programming in ATN DBS, and provides supportive evidence for future prospective trials using the ATN-MMT connection as a direct surgical target.

#### Closed-loop systems

The core principle of a closed-loop system is responsive neural stimulation, which integrates epilepsy seizure detection and prediction algorithms along with stimulation devices. This approach uses real-time monitoring and automatic response mechanisms to control epileptic seizures [204]. Closed-loop systems continuously monitor the brain’s electrical activity through implanted electrodes or external sensors. Upon detecting signs of an impending seizure, the system automatically triggers a preset response, such as electrical stimulation, to prevent or mitigate the episode. Compared to open-loop stimulation, closed-loop systems activate only when seizure indicators are detected, thus reducing unnecessary stimulation and potentially minimizing long-term side effects. In line with this concept, Kassiri et al. [205] introduced a 64-channel closed-loop neural stimulator system-on-a-chip, validated in vivo for both epilepsy monitoring (seizure detection) and treatment (seizure suppression). Further advancing the field, Campos-Rodriguez et al. [206] investigated the inhibitory effect of optogenetic stimulation of the superior colliculus on spontaneous seizures in a genetically absent epilepsy model. The results indicate that single-cell activity in the deep and intermediate layers of the superior colliculus significantly decreases a few seconds before the onset of pulse wave discharges while markedly increasing during and after seizures. Nearly 40% of neurons exhibit inhibitory firing at the onset of pulse wave discharges. Continuous optogenetic stimulation of the deep and intermediate layers of the superior colliculus significantly reduces pulse wave discharges in males but has no effect on females. Conversely, closed-loop neuromodulation is effective for both males and females. Moreover, van Blooijs et al. [207] pioneered the use of single-pulse electrical stimulation to identify potentially effective cortical networks and select a stimulation site connected to the epileptic focus. In the experiment, one subdural strip was implanted above the epileptic focus, while another was positioned at the selected stimulation site. A subcutaneous neurostimulator, capable of recording and closed-loop stimulation, was connected to both strips. Initially, a 3 − 5-month data collection period was conducted to optimize the epilepsy monitoring algorithm. Subsequently, closed-loop electrical stimulation was implemented over the next 7 − 9 months. Clinical trial results indicated that in the 5 subjects with refractory epilepsy arising from the motor cortex, closed-loop electrical stimulation significantly reduced seizure frequency in all participants. In addition to these findings, Kobayashi et al. [204] conducted a retrospective study on 12 patients who received cortical evoked potentials during stereoelectroencephalogram and subsequently underwent responsive neurostimulation therapy. They found that functional connectivity determined by cortical-to-cortical evoked potentials may provide additional information to guide the optimal placement of responsive neurostimulation electrodes. Furthermore, Anderson et al. [208] investigated the impact of closed-loop stimulation during periods of low epileptic activity on improving the therapeutic effect of epilepsy treatment. The study hypothesized that stimulation during low-risk states may help restore healthy brain networks and reduce long-term seizure frequency.

In the past few decades, neurostimulation has emerged as a crucial treatment option for patients with refractory epilepsy. Through DBS and vagus nerve stimulation, research has found that large-scale brain networks can be modulated, leading to a reduction of seizure frequency by approximately 50% in about half of the patients. With the continuous advancement of neuroscience and technology, the mechanisms underlying neurostimulation will become increasingly clear. Furthermore, future neurostimulation devices are expected to become more intelligent and miniaturized, capable of automatically adjusting stimulation parameters or triggering neuromodulation responses upon detecting early signs of seizures, thereby more effectively preventing seizure occurrence.

### BCIs in cerebral resuscitation

Post-cardiac arrest brain injury is one of the significant causes of death for patients who have recovered spontaneous circulation after cardiopulmonary resuscitation following cardiac arrest. It is also the primary reason for long-term functional disabilities among survivors in the acute phase. Cerebral resuscitation, an umbrella term for post-recovered spontaneous circulation brain protection treatments, aims to rapidly implement rescue measures to restore circulatory and respiratory functions, ultimately leading to the recovery of patient consciousness [209]. The primary goal is to achieve adequate brain tissue oxygenation, minimize the degree of brain injury, and promote the return of brain function to a normal state [210]. Currently, standard arousal methods include electrical stimulation therapy, magnetic stimulation therapy, hyperbaric oxygen therapy, and mild hypothermia therapy. However, even if a patient’s heartbeat and breathing are restored, their consciousness may not fully recover, and they may fall into a coma or vegetative state. In such cases, communicating with patients, understanding their needs, and assessing their consciousness status pose significant challenges for the medical community. In recent years, the development of BCIs has provided new possibilities for addressing these challenges. BCIs allow for the collection and analysis of patients’ brain signals to detect whether they can follow commands, thereby assessing their consciousness status [211]. Therefore, BCIs exhibit significant application potential in areas such as the arousal of comatose patients and communication with patients in a vegetative state.

#### Diagnosis and evaluation of consciousness disorders

The Department of Neurosurgery, Beijing Tiantan Hospital successfully evaluated the consciousness status of a female patient who had been in a coma for 4 months after spontaneous cerebral hemorrhage surgery using BCI technology combined with functional MRI, EEG, and ERP [212]. After evaluating the patient’s brain function using EEG and ERP, it was determined that the patient exhibited good brain function and a high level of consciousness. Additionally, SSVEP-based BCI typing technology was employed, allowing patients to provide answers on a screen through output or selection without requiring verbal or physical actions; instead, it relies solely on the observation and imagination of corresponding options. This method provides a reliable scientific basis for subsequent diagnosis and treatment. Furthermore, Pan et al. [213] proposed an audio-visual hybrid BCI based on auditory P300 and SSVEP. The results demonstrated that 4 healthy subjects, 1 patient in a vegetative state, 1 patient in a minimally conscious state, and 1 patient with LIS successfully completed the intended tasks. Similarly, Qin et al. [214] established a quantitative prognostic prediction model based on EEG for non-traumatic coma patients, which can monitor the state of consciousness and guide clinical practice.

#### Enhancing functional recovery in aroused patients

For patients who have regained consciousness after treatment, even to a normal level, but are unable to express their consciousness due to severe impairment of language and motor functions, detecting and successfully outputting their conscious state remains a significant clinical challenge. BCIs and other human–machine interaction methods have emerged as promising solutions to this issue [212].

For patients with aphasia or speech difficulties due to brain injury, BCIs can classify and recognize brain signals by identifying specific patterns of EEG activity. This enables patients to control computer input devices such as spellers and speech synthesizers using their thoughts, thereby facilitating communication with the outside world. Spellers convert patients’ intentions into text output by recognizing their brain signals, while speech synthesizers decode neural activity related to language in the brain to generate voice output [215]. The application of BCI technologies, including spellers and speech synthesizers, has enabled aphasic patients to re-establish effective communication with the outside world, thus enhancing their social interactions and quality of life.

For patients with motor dysfunction resulting from brain injury, BCIs can assist in partially restoring motor abilities and improving quality of life. Recent research has shown that BCI-triggered FES therapy successfully restored upper limb function in a 57-year-old male with severe left hemiplegia [216]. Additionally, research has demonstrated that incorporating the MI paradigm into exoskeleton assistive devices can further enhance rehabilitation outcomes following stroke [217]. BCI technology not only facilitates rehabilitation training but also provides new insights into neural mechanism research. By monitoring and analyzing brain signals in real-time, we can gain a deeper understanding of the functions and mechanisms of the nervous system, thereby providing theoretical support for research on neural function recovery. A previous study has indicated that reorganizing brain functional network topology after BCI training increases coordination between multisensory and motor-related cortices and the extrapyramidal system [218].

The application of BCIs in cerebral resuscitation holds broad prospects and significant potential. BCI technology provides robust technical support for brain resuscitation by facilitating the diagnosis and evaluation of consciousness disorders, promoting neural rehabilitation and functional recovery, and improving communication abilities. In the future, with ongoing technological advancements and expanding clinical applications, BCIs are expected to play an increasingly important role in the field of brain resuscitation, thereby benefiting a greater number of patients.

### BCIs in sleeping

Sleep is a complex physiological process that plays a key role in an individual’s cognitive function, development, and recovery [219]. Sleep-related disorders have a profound impact on an individual’s health and significantly reduce quality of life. Poor sleep quality can lead to obesity, diabetes, cardiovascular disease, hypertension, mood disorders, and impaired immune system function [220–224]. With the development of BCI technology, real-time sleep monitoring and sleep regulation systems using open- and closed-loop stimulation techniques are gradually becoming a focal area of research.

#### Sleep detection

The diagnosis of sleep disorders and the subsequent implementation of closed-loop sleep regulation systems depend on the development of advanced sleep detection techniques. These technologies primarily rely on analyzing polysomnographic recordings throughout the night. Manual scoring considered the gold standard for sleep analysis, is a time-consuming task that can only be performed by trained experts [225]. With the advancement of AI, the speed and accuracy of sleep EEG analysis have been significantly enhanced. Traditional sleep EEG classification methods typically involve two steps: features are extracted from preprocessed EEG signals, and then, sleep stage classifiers are constructed. In these traditional methods, many features are designed based on established sleep knowledge [226]. In recent years, with the rapid development of DL, researchers have shown increasing interest in algorithms that can automatically learn features. DL algorithms enable end-to-end classification and extract the most representative features from large datasets without relying heavily on a priori knowledge, offering specific advantages over traditional algorithms. Consequently, an increasing number of sleep EEG analysis models based on CNNs [227], LSTMs [228], transformers [229], GCN [230], and other architectures have been reported. For example, Yao et al. [229] proposed a vision-transformer-based architecture for processing multichannel sleep map signals. The model captures spatial information from multiple polysomnographic channels using a vision transformer and employs a self-attention mechanism to understand transitions between adjacent periods. Additionally, the authors utilized pre-trained weights from a large image dataset to address the issue of insufficient data. In a related advancement, Kong et al. [226] introduced a novel neural architecture search framework based on a two-layer optimized approximation for EEG-based sleep stage classification (SSC), offering guidance for the automatic design of subsequent sleep classification networks. Furthermore, Eldele et al. [231] conducted an in-depth analysis of 3 SSC datasets. They found that fine-tuning a pre-trained SSC model with only 5% labeled data can achieve performance comparable to fully labeled supervised training. Moreover, self-supervised pre-training improves the robustness of SSC models against data imbalance and domain shift issues. Nonetheless, the sleep EEG analysis domain continues to face numerous challenges, necessitating innovative solutions. Firstly, reducing the computational complexity and energy consumption of models while maintaining high accuracy remains an urgent problem. Secondly, the high individual variability of sleep data and the significant differences in sleep EEG features among individuals place higher demands on the model’s generalization ability. Therefore, future research should focus on developing more efficient and robust sleep EEG analysis models to facilitate the development of sleep disorder diagnosis and regulation techniques.

#### Sleep regulation

Sleep regulation is considered a crucial approach to studying and enhancing the mechanisms of sleep. The primary objective is to modulate sleep through the introduction of auditory, olfactory, visual, and other stimuli. Sleep regulation based on BCIs can be primarily categorized into two forms: open- and closed-loop systems. In traditional stimulus regulation experiments, the stimuli were based on preset parameters independent of brain activity. This conventional method of investigating causal phenomena makes it challenging to accurately elucidate the connection between stimulus outcomes and underlying brain mechanisms. With advancements in computer technology, researchers can now analyze brain activity in real-time and develop closed-loop systems that provide targeted stimulation of specific sleep components based on brain activity. However, both types of systems have their respective advantages and disadvantages and are suited to different research directions.

##### Open-loop systems

Open-loop stimulation methods are mainly used to elucidate sleep characteristics. For example, Kande et al. [232] induced sleep spindle waves by applying peak stimulation to the rat thalamus, revealing the mechanisms and roles of sleep spindle waves. In another study, Prehn-Kristensen et al. [233] employed transcranial oscillating direct current stimulation (DCS) at a frequency of 0.75 Hz to investigate whether externally triggered enhancement of slow-wave oscillations during early slow-wave sleep could improve memory performance in children with attention deficit hyperactivity disorder. The findings have demonstrated that augmenting the power of slow oscillations during sleep via DCS can mitigate declarative memory deficits in children with attention deficit hyperactivity disorder. Similarly, Saebipour et al. [234] applied slow (0.75 Hz) oscillatory transcranial DCS (tDCS) during the second stage of non-rapid eye movement in patients with insomnia, aiming to synchronize the patient’s brainwaves with the frequency of sleep slow waves. Six patients with insomnia participated in this study, and the results showed that the corresponding stimulation intervention had a sleep-stabilizing effect, potentially mimicking the effects of sleep slow-wave-enhancing medication. Additionally, studies of sound stimulation for sleep regulation have also been reported. Bellesi et al. [235] investigated the physiological mechanisms of the K-complex, a peripherally induced slow wave. They demonstrated that acoustic stimulation was the most effective in increasing the amplitude of slow waves across different sensory modalities. Studies have also examined how the intensity, frequency, timing, and mode of acoustic stimulation can influence sleep enhancement. Ngo et al. [236] reported enhanced slow wave activity during sleep stages and relatively deeper sleep by applying tonal stimulation to subjects during sleep. Moreover, numerous studies have documented the effects of olfactory sensory stimulation [237] and tactile sensory stimulation [238] on sleep stabilization.

##### Closed-loop systems

With advances in computer technology, there has been a significant increase in research on closed-loop systems for sleep regulation. This shift is driven by the limitations of open-loop systems in providing a comprehensive understanding of sleep mechanisms [239]. The closed-loop stimulation paradigm establishes a continuous feedback loop between neural circuitry and the external environment. It involves controlling the external environment based on real-time neurophysiological information and providing targeted feedback to the subject, thereby influencing their brain activity [240]. For example, Lustenberger et al. [241] selectively enhanced human sleep spindle waves using non-invasive alternating current stimulation, demonstrating that this targeted modulation significantly improved motor memory consolidation and provided direct evidence for the relationship between spindle activity and cognitive function. Similarly, Santostasi et al. [242] employed a phase-locked loop to deliver acoustic stimuli at specific phases of electrophysiological rhythms, thus enhancing slow-wave sleep through periodic acoustic stimulation. Besides, Ketz et al. [243] aimed to augment slow-wave oscillations using a closed-loop transcranial alternating current stimulation system. They reported enhanced subjective sleep quality following closed-loop transcranial alternating current stimulation and improved long-term memory consolidation. Moreover, Ngo et al. [244] reported that delivering exogenous stimuli at the optimal phase of endogenous brain rhythms can improve experimental methods for enhancing memory during sleep.

The effectiveness of closed-loop stimulation systems in promoting sleep quality has spurred interest in portable and wearable devices. In this context, Ferster et al. [245] introduced a dynamic sleep-mobile system capable of monitoring sleep biosignals and providing real-time interventions. The authors revealed that their device is suitable for unobtrusive multi-night monitoring and intervention in home settings. Similarly, Lu et al. [246] proposed a closed-loop auditory stimulation system for sleep regulation, leveraging machine learning to track sleep stages and provide rapid feedback via real-time automated sleep staging online. This system offers continuous external auditory stimulation during specific slow-wave phases. Additionally, Bressler et al. [247] designed a wearable EEG device enabling closed-loop real-time tracking and neuromodulation of various sleep-related oscillations. Preliminary results indicate that this approach can offer non-invasive neuromodulation across all sleep stages.

Future research should focus on refining algorithms and techniques for closed-loop systems to enhance the precise modulation of specific sleep components, thereby further elevating sleep quality and cognitive function. Concurrently, as portable and wearable technologies continue to advance, these systems could become more accessible and user-friendly, paving the way for personalized sleep regulation solutions.

### BCIs in the diagnosis and treatment of neurodegenerative diseases

#### Diagnosis and treatment of AD

AD is currently the most common cause of dementia globally, accounting for approximately two-thirds of cases worldwide [248]. The primary symptoms include a decline in cognitive function and changes in behavioral patterns, which can ultimately lead to death in severe cases [249]. According to recent statistics, there are approximately 55 million people worldwide suffering from AD in 2023, with this number doubling every 5 years. By 2050, the number of patients is estimated to reach around 150 million [250]. Current treatment methods for AD include cholinesterase inhibitors, other psychiatric medications, and conservative treatments; however, none of these approaches can completely cure the disease. Recent research has shown that BCIs can effectively assist in diagnosing AD and may help alleviate its progression through specific training methods [251–263].

AD typically exhibits no obvious clinical symptoms in its early stages, making this period the optimal time for intervention. BCI technology can detect early changes in brain function, assisting in early diagnosis and timely intervention. For instance, Fukushima et al. [251] applied a character-based BCI to detect AD by asking participants to focus on specific characters. They successfully distinguished AD patients from those with mild cognitive impairment by analyzing the P300 ERP of the participants’ gaze, achieving an accuracy of up to 80%. Similarly, Ung et al. [252] combined fNIRS with BCIs to adjust task difficulty in real-time based on fNIRS signals. The results showed that the healthy control group could perform higher task levels, indicating the potential of fNIRS-BCI as a diagnostic tool for detecting cognitive function in AD patients. Further advancing this approach, Kang et al. [253] optimized the diagnostic method of fNIRS-BCI by proposing a new multi-classification model named CNN-LSTM, which effectively diagnosed early-stage AD patients with an average accuracy of 77.77%, demonstrating the excellent potential of fNIRS-BCI in detecting AD.

Treatments for AD based on BCI technology have also garnered significant attention from researchers. These interventions operate through various mechanisms, including enhancing neuroplasticity, regulating neuroinflammation, and improving cerebral blood flow [254]. Among these, exercise intervention is particularly crucial, as research has shown it can significantly enhance cognitive function in patients with AD. These benefits are primarily attributed to increased cerebral blood flow, improved cardiovascular health, and the modulation of vascular factors [255]. Furthermore, exercise can activate signaling pathways such as Wnt and PI3K/Akt, aiding in alleviating autophagy dysfunction and reducing amyloid-beta deposition [256]. Exercise also enhances antioxidant capacity and regulates immune responses, thereby decreasing tau protein and cortisol levels [257]. Integrating BCI technology into exercise rehabilitation provides patients with greater autonomy and a sense of participation, further enhancing rehabilitation outcomes. Galvin-McLaughlin et al. [258] proposed a combination of BCI and neurofeedback (NFB), where subjects were trained using three paradigms: finding the indicated letters, copying spelled words, and crossing out the target letters. It was found that BCI-based NFB intervention for AD is practically feasible but requires further validation due to the limited number of participants. In another study, Lin et al. [259] observed a significant increase in frontal γ coherence through NFB training in AD patients. Moreover, the baseline F4 and pre-frontal γ power levels could serve as predictive factors for training effectiveness, further confirming the potential of combining NFB and BCI for AD rehabilitation. Additionally, White et al. [260] pioneered the use of VR for rehabilitating AD patients by creating a symmetrical, landmark-free house model in VR and training patients to reach designated locations. The 7-week training results showed significant improvements in cognitive function. Although White et al. [260] did not combine VR with BCI, this approach provided a novel method for treating AD. Building on this, Leeb et al. [261] proposed integrating BCI and VR to stimulate the brain through multisensory means for rehabilitating neurodegenerative diseases. Jiang et al. [262] developed a VR-BCI system that assisted clinical treatment of AD by repeatedly stimulating memory. Lin et al. [263] also integrated VR with BCI by using 4 games, Schulte Grid, Eureka Effect, Stroop Test, and Soma Cube, to monitor real-time EEG signals in the α, β, θ, δ, and γ bands for the treatment of AD and cognitive impairment.

Although BCIs have made significant progress in the diagnosis and treatment of AD, they still face several substantial challenges. One notable issue is that patients with advanced disease progression may be unable to effectively control their brain activity. Therefore, how to incorporate BCIs into the diagnosis and treatment of AD remains an important direction for future research.

#### Diagnosis and treatment of PD

PD is the second most common neurodegenerative disease worldwide, with its incidence increasing with age. The disease is chronic and progressive, clinically characterized by 4 primary symptoms: resting tremor, muscle rigidity, bradykinesia (slowness of movement), and postural instability. Pathologically, it is marked by degeneration of the dopaminergic system in the substantia nigra pars compacta of the midbrain and the presence of characteristic inclusions known as Lewy bodies in the remaining neurons. With the advancement of modern pathological techniques, it has been discovered that the pathological changes in PD are not confined to the substantia nigra pars compacta but involve the extensive nervous system throughout the body. Currently, the diagnosis of PD primarily relies on the patient’s medical history and clinical examination [264]. Diagnosis requires the presence of motor symptoms (such as bradykinesia, tremor, and postural instability) and a positive response to traditional anti-PD medications while excluding secondary causes of PD (such as head injury or exposure to toxic substances). In reality, before the onset of motor symptoms, more than 50% of the substantia nigra neurons have already degenerated [265]. By the time of diagnosis, the disease is often at an advanced pathological stage. If PD could be diagnosed and intervention initiated before the clinical symptoms appear, it would open a new chapter in the treatment of PD.

EEG signals contain a wealth of physiological and pathological information, which change correspondingly when neurogenic, pathological, or functional alterations occur within brain tissue. Therefore, in clinical medicine, analyzing EEG signals can provide a basis for diagnosing and treating brain diseases. In EEG recordings from the basal ganglia region, PD is characterized by pathological oscillations in the β frequency band (14 − 30 Hz) [266], with β power being associated with the severity of PD symptoms [267]. Additionally, emerging theories suggest that PD is marked by excessive synchronization in the β frequency band (around 20 Hz) throughout the entire basal ganglia-thalamo-cortical circuit. Swann et al. [268] identified a standard for measuring this synchronization using invasive EEG recordings: phase-amplitude coupling (PAC) between β phase and broadband γ amplitude. This standard was also validated using non-invasive EEG, showing that PAC was higher in patients who had paused their medication compared to those on medication and healthy controls. This indicates that EEG PAC could serve as a non-invasive biomarker for diagnosing PD.

Implantable BCIs with recording and stimulation functions have revolutionized the prognosis and treatment of PD. Continuous detection of neural signals allows for the developing of more accurate and personalized treatment plans. Therapeutic electrical stimulation induces alternations in brain activity by delivering electrical pulses to targeted brain areas. DBS can be adjusted according to the patient’s symptoms and targets various nodes of the basal ganglia-thalamo-cortical circuits mediating different disease symptoms. Specifically, DBS in the thalamus is most effective for tremors, DBS in the globus pallidus is most effective for rigidity and dyskinesia, and DBS in the subthalamic nucleus can treat tremor, akinesia, rigidity, and dyskinesia [269]. The improvement in PD symptoms with DBS is significant. Olchik et al. [270] evaluated the quality of life of PD patients before and after DBS surgery and found an improvement in overall quality of life scores post-DBS. In addition to traditional DBS surgery, researchers use DBS electrodes to collect local field potentials and then employ BCIs to assess and provide feedback electrical stimulation, known as adaptive DBS. Adaptive DBS has shown further improvements in patients’ motor scores [271].

Additionally, BCIs play an important role in the rehabilitation of PD patients. Benninger et al. [272] explored the efficacy of anodal tDCS applied to the motor and prefrontal cortex. Their research found that tDCS can improve specific gait parameters in the short term and reduce bradykinesia in both the “on” and “off” states for over 3 months.

### BCIs in anesthesia

General anesthesia is designed to block the patient’s motor responses and inhibit autonomic responses to injurious stimuli [273]. It can also be viewed as a safe and reversible loss of consciousness induced by an anesthesiologist through the administration of anesthetic agents [274]. The emergence of general anesthesia has alleviated patients’ fears associated with imagining surgery without anesthesia. For surgeons, general anesthesia suppresses the patient’s involuntary response to noxious stimuli, ensuring a smooth and controlled surgical procedure.

#### Significance of anesthetic depth monitoring

Anesthesia not only effectively alleviates pain and ensures patient comfort during surgical procedures but also induces a state of unconsciousness, thereby reducing fear and anxiety related to the surgery. However, the dosage of anesthetic has significant effects on patients during general anesthesia. Insufficient depth of anesthesia can lead to intraoperative awareness, while excessive depth may result in delayed recovery, postoperative delirium, cognitive problems, and other adverse reactions [273]. Monitoring the depth of anesthesia is crucial as it provides anesthesiologists with an objective and accurate assessment of the patient’s state of consciousness during surgery, assisting them in controlling the anesthetic dosage to prevent intraoperative awareness and minimize postoperative complications [275]. The ideal anesthesia depth monitoring equipment should possess the following functions: 1) accurately measure the patient’s sedation state; 2) be simple and convenient for clinicians to install and use; 3) provide clinically relevant information; and 4) offer sensitive, stable, and interference-resistant data monitoring. Anesthesia depth monitoring based on EEG provides a novel approach to directly reflect whole-brain consciousness changes, providing excellent potential for comprehensive and individualized anesthesia detection.

#### The BCI-based anesthesia depth monitoring

The bispectral index (BIS) is widely used to monitor and assess the depth of anesthesia in patients, ensuring safety and patient comfort during surgical procedures [276]. The BIS monitors changes in EEG signals in the prefrontal region and produces a standardized index ranging from 0 to 100. This index reflects brain activity: higher BlS values indicate greater patient consciousness, while lower BIS values indicate deeper levels of unconsciousness [277]. Generally, a BIS value of 80 − 100 indicates wakefulness, 60 − 80 indicates moderate sedation with no response to mild stimuli but responsiveness to severe stimuli, and 40 − 60 indicates unconsciousness. This range is considered optimal for surgery as it ensures that the patient’s brain is not overly affected by excessive anesthetic drugs, nor will the patient wake up due to surgical pain. A BIS value of 20 − 40 indicates deep sedation and 0 − 20 indicates burst suppression, where patients do not respond to external stimuli, but excessive anesthesia depth can impair involuntary functions such as breathing and heartbeat, potentially leading to serious complications like respiratory arrest. During a typical anesthesia process, a patient’s BIS rapidly drops to 40 − 60 upon induction and remains within this range throughout the operation. After surgery, anesthesia administration ceases, allowing the patient’s consciousness to gradually recover, with the BIS value slowly returning to normal.

The BIS monitor is the first depth of anesthesia monitor approved by the Food and Drug Administration to assess the effectiveness of clinical anesthesia. As the pioneering and most extensively studied EEG-based anesthesia depth monitor, BIS features a simple and convenient application (requiring only 4 electrodes placed on the frontal lobe), stable performance across multiple anesthetics and their combinations, and has set a benchmark for the development of future EEG monitors. The technical maturity of BIS monitors provides robust support for guiding anesthesia in domestic and international intensive care units [278]. The cerebra state monitor (CSM) is a battery-powered portable device used to detect anesthesia depth. CSM employs a fuzzy logic algorithm to analyze the relationship between parameters derived from collected EEG data and the brain state index, thereby evaluating the patients’ anesthesia depth. Similar to the BIS index, the CSM index ranges from 0 to 100 [279].

The Narcotrend EEG monitor is one of the most commonly used and widely recognized anesthesia depth monitoring instruments in China. A significant distinction from other EEG-based anesthesia depth monitors is that it not only provides parameter indicators (Narcotrend indicators) with a range of 0 − 100 for assessing patients’ consciousness but also subdivides this range into 6 grades, labeled A to F [280]. Additionally, the monitor displays a heart rate, 95% spectrum edge frequency, and power spectrum information for various EEG frequency bands (a, β, γ, etc.), providing users with comprehensive data for observation and analysis. Relevant clinical study has shown a high correlation between the Narcotrend monitor and the BIS monitor when using propofol and certain volatile anesthetics [279].

SEDLine is one of the few EEG-based anesthesia depth monitors that has undergone extensive updates in recent years. Several studies have indicated that the new algorithm of SEDLine can effectively improve its parameter index and may exhibit notable differences from the BIS index in clinical practice [281, 282]. SEDLine innovatively employs a 5-frontal electrode placed on the frontal region to record 4-channel EEGs. Instead of processing a single hemisphere, it simultaneously processes both hemispheres to generate a density spectral array, allowing for the observation of power spectrum symmetry changes between the left and right hemispheres. Clinical evidence suggests that patient age should be considered when using the EEG state index to avoid administering excessive anesthetic drugs to elderly patients [282].

Contemporary EEG monitors have achieved remarkable success in monitoring the depth of anesthesia, but the influence of different drugs and patient ages on their monitoring accuracy remains a challenge. Further research is needed to refine EEG-based anesthesia depth detection methods.

### Others

An essential application of BCIs is the continuous monitoring of brain activity, which can be used to assess various aspects of cognitive state, including emotion recognition, cognitive load assessment, and attention detection.

#### BCIs in emotion recognition

Physiological signals such as EEG and cerebral blood flow can reflect the emotional state of individuals [283]. Consequently, these signals can detect changes in anxiety, stress, interest, and other emotions, with applications in mental health assessment and user experience optimization. Qi et al. [284] investigated the difference in EEG features between patients with high generalized anxiety disorder (HGAD) and patients with low generalized anxiety disorder (LGAD). They found that the phase lag index values for α rhythms significantly increased in HGAD patients, while θ and α1 rhythms were significantly reduced. Additionally, the small-world property was less than 1 in both HGAD and LGAD patients. Furthermore, the θ and α rhythm values in HGAD patients were significantly lower than those in LGAD patients, indicating that the brain’s functional network structure deteriorates as the severity of generalized anxiety disorder increases. In another study, Zhou et al. [285] developed a DL model based on EEG features to simulate internal EEG information and accurately predict human state perception. This has practical significance for alerting subjects to release emotional stress and alleviate anxiety. Interest is an important emotional characteristic. Aminiroshan et al. [286] found that the electrical attention index differs significantly between interested and non-interested individuals while watching videos.

Emotional changes significantly impact mental health, which has become increasingly important in contemporary society. For example, Luo et al. [287] found that mindfulness training can improve college students’ mental health, as evidenced by changes in the α band in the frontal lobe region and θ band in the midline region on EEG. This provides an objective method for assessing mental health. Furthermore, changes in the state of consciousness significantly influence a person’s behavior. Niu et al. [288] developed an explainable model to evaluate the quality of video service experience. The model integrates single- and multi-electrode features to identify sensitive regions, thereby optimizing and developing video services as well as providing a scientific basis for improvement.

#### BCIs in cognitive load assessment

Humans generate a substantial cognitive load when performing complex tasks. Liu et al. [289] designed a novel paradigm for predicting cognitive load levels by integrating spatial, temporal, and spectral EEG features. The fusion of these three types of features enhances prediction performance, and each type can independently serve as a feature for cognitive load assessment. Additionally, the study also shows that an increase in cognitive load leads to elevated θ band rhythm and reduced α band rhythm, resulting in significant changes in connectivity between related channels and microstates.

#### BCIs in attention detection

BCIs can detect real-time attention levels to assess work efficiency and learning outcomes. In tasks requiring high concentration, attention detection can alert operators to decreased attention, thereby enhancing safety. Rabbani et al. [290] have studied human attention parameters using statistical indicators, developing an EEG signal prediction system that leverages EEG analysis to detect human attention levels. This system is expected to effectively monitor subjects’ attention states.

BCIs provide a novel tool and method for cognitive assessment and demonstrate significant potential in advancing cognitive neuroscience in various application domains. As technology continues to progress, the prospects for applying BCIs in cognitive detection are becoming increasingly promising.

## Current challenges and future trends

With the rapid advancement of human–computer interaction and the development of AI algorithms, BCIs have found extensive applications in the medical field, particularly fo patients with brain disorders. However, significant challenges remain before this technology can be widely adopted by the public.

### Current challenges in BCIs

Inevitably, the rapid development of technology brings with it a series of challenges that require addressing. Truly user-friendly technologies are essential to enhance people’s lives and ensure convenience, versatility, privacy, and security. Before deploying BCI technologies in the community, researchers and practitioners must engage users and ensure that the technology meets predefined quality standards.

#### Personal privacy and ethical challenges in BCIs

Ethical and privacy issues are critical to the development of BCI technologies [81]. Privacy can be defined as the right to prevent unauthorized access to personal information and personal space [291]. Firstly, brain data, which encompasses a wide range of information recorded from neural activity, can provide highly personal insights closely related to an individual’s identity [292]. To some extent, individuals can control the facial expressions and images they present to the outside world. However, unintentional behaviors may inadvertently reveal some information. Collecting brain data could offer new avenues for transcending this limited control [291], potentially compromising strategies such as hiding unsanctioned emotions that individuals use to preserve privacy. Furthermore, brain data may contain sensitive information about neurological conditions, which could be revealed without explicit consent. Secondly, in recent years, personal privacy has faced unprecedented challenges. The commercial sector has achieved an unparalleled capacity to acquire and analyze personal information. Social media companies can examine users’ social, political, religious, and consumer behaviors, using or exchanging this data for targeted information dissemination [293]. Brain data represents some of the most sensitive and private information individuals possess; without adequate regulation, their privacy may no longer be adequately protected. Thirdly, brain data is one of the few areas that have not yet experienced significant privacy violations. While it may be too late to restrict the collection of location data, video surveillance, commercial preferences, and behavioral data, ubiquitous brain-recording devices have not yet emerged. However, this situation could change as substantial commercial capital flows into the development of consumer neurotechnology [294]. Therefore, safeguarding the privacy of EEG signals must be a top priority.

#### Network attack and vulnerabilities in BCIs

BCIs have made significant progress in medical applications and product development. However, security concerns have become increasingly prominent with the growing demand for internet communication in BCIs. The widespread adoption of BCI technologies exposes these systems to potential cyber-attacks, which can disrupt their normal operation [295]. Attackers may alter the instructions issued by feature transformation components, potentially causing adverse effects on target individuals [296]. Therefore, researchers should thoroughly investigate the security threats and vulnerabilities within BCI systems and develop practical solutions to enhance system security.

#### Safety concerns and biocompatibility in BCIs

Safety issues are a significant concern in the research and development of invasive BCIs. When these devices are implanted into brain tissue, they may cause damage to neurons and blood vessels, increasing the risk of infection [297]. Furthermore, the body’s immune system may recognize the implant as a foreign substance, leading to biocompatibility issues [298]. Additionally, scar tissue formed after surgery may gradually degrade the quality of brain signals [299]. To address these challenges, it is essential to conduct in-depth research on how the human body functions and its response to foreign materials is essential. BCI researchers and engineers should leverage this knowledge to develop safer and more efficient applications. Moreover, this information should guide neurosurgeons in achieving more precise implantation of BCI electrodes in specific brain regions.

#### User acceptance in BCIs

Various factors influence patient and user acceptance of BCI technologies. BCI research should extend beyond detection performance, speed, and bit rate. While these metrics are important, they represent only a fraction of the broader spectrum of user needs. Studies indicate that there is only partial overlap between the evaluations of BCI technologies by potential users and experts [300–304]. Therefore, adopting user-centered design and experimental paradigms in human–computer interaction research is essential to enhance user experience and meet the specific needs of different user groups. Furthermore, before deploying BCI technologies in community settings, researchers and practitioners are responsible for engaging users and ensuring that the technology meets predefined quality standards.

Device comfort and naturalness are also critical factors. Most EEG-based BCI systems currently comprise electrodes, amplifiers, and accessories (e.g., head caps). The design and wearing experience of these systems directly affect their potential application scenarios. To address this challenge, portable and lightweight EEG devices have emerged. However, such portable devices still require electrode housings and fixation via headbands to ensure compactness and ease of wear [303]. Nonetheless, persuading healthy individuals to wear uncomfortable and aesthetically unappealing EEG caps or headbands in daily life remains a significant challenge, especially given that current BCI systems do not yet offer enough functionality to substantially improve quality of life. Therefore, developing more comfortable, natural, and stylish devices is imperative.

#### Signal acquisition quality and stability in BCIs

Noise interference and signal attenuation significantly impact the quality of EEG signal acquisition. Non-invasive devices are particularly susceptible to signal fading during transmission through the scalp and skull [29]. Thus, researchers need to optimize signal extraction at both hardware and algorithmic levels to enhance subsequent research and analysis. Implantable devices offer more accurate localized EEG signals. However, they present challenges related to long-term stability, biocompatibility, and durability, including preventing displacement, infection, and material deterioration.

#### Limitations of data analysis

Signal decoding is the most critical step in connecting the brain with machines in BCIs. While most decoding algorithms can achieve promising performance under ideal conditions, several real-world challenges remain to be addressed.

##### Limited training samples

A large and high-quality dataset is essential for advancing DL networks. However, acquiring a substantial amount of high-quality data poses significant challenges. Data augmentation techniques can enhance the quantity and diversity of training samples. Nevertheless, these methods based on transformations of existing samples often fail to accurately simulate the complex distribution of actual EEG signals [304]. Therefore, a critical challenge is to delve into the underlying patterns present in EEG signals to generate physically meaningful simulated samples.

##### Distribution mismatch

The distribution of training data may significantly differ from that encountered in actual applications, often due to variations in experimental environments or populations [305]. While knowledge transferred from other users can aid in model training for the target user, such discrepancies can lead to distribution mismatch issues. Therefore, enhancing the model’s generalization ability to new distributions through domain adaptation methods in transfer learning is essential.

##### Data calibration

Most BCI applications require data calibration to correct for undesirable changes caused by neuroplasticity or micromotion of the electrode array [297]. This process is often inconvenient and time-consuming, imposing an additional burden on users. Future research should focus on developing automated calibration methods to streamline operational updates of BCI devices, thereby improving user experience.

### Future trends in BCIs

The above challenges urgently need to be addressed to facilitate the development and application of BCIs in the medical field. Based on these challenges and the current state of research, we propose the following future trends in BCIs.

#### Multimodal-based BCI technology

While EEG-based BCIs are the most widely studied and applied due to their non-invasive nature, affordability, and portability, other BCI modalities also hold significant potential for advancing the field. For instance, MEG provides high temporal and spatial resolution, making it suitable for studying deeper brain activity and complex neural networks. Although its cost and lack of portability currently limit its widespread use, future developments in compact and affordable MEG devices could enhance its accessibility and application in real-world scenarios. Similarly, fNIRS offers a non-invasive approach to measuring brain activity through the hemodynamic responses. Its high compatibility with wearable systems and resistance to electrical noise make it a promising complement to EEG, particularly in hybrid systems that combine signals for improved accuracy and reliability. Integrating these modalities with EEG in hybrid BCIs could leverage their respective strengths, enhancing system performance and expanding applications in fields such as healthcare and medical treatment. Future research should explore these synergies and address the challenges of multi-modal integration, paving the way for more versatile and practical BCIs.

#### Optimization of EEG paradigms and algorithms

EEG experiments involve paradigm design, metric evaluation, and algorithm development. Therefore, these three aspects are crucial to developing user-friendly BCI applications.

##### Designing more natural BCI paradigms

Traditional BCI paradigms often make human–computer interaction seem unnatural and static, requiring significant cognitive resources to perform actions. Researchers should therefore focus on developing new, more natural BCI paradigms that significantly reduce the cognitive load on users. This will enhance the intuitiveness and fluidity of BCI system usage.

##### New metrics for evaluating BCI interactions

While algorithmic accuracy and information transfer rates are important, evaluating BCI interactions in real and complex human–computer interaction scenarios requires considering human factors as well. This includes effectively integrating user feedback and experience into closed-loop operations to comprehensively assess the actual performance and application effects of the BCI system [303].

##### More efficient algorithmic processing and data analysis

Algorithms are a critical component of EEG signal decoding, but developing algorithms without a thorough understanding of EEG mechanisms is not advisable. A deep understanding of EEG mechanisms will significantly guide the design of BCI algorithms. Additionally, individual variability in EEG signals limits the reproducibility and generalizability of BCIs, thereby reducing the robustness of brain decoding algorithms. Advanced BCI algorithms need to minimize both intra- and inter-individual variability to create a more reliable BCI system. Investigating the neural mechanisms underlying these EEG variabilities is a promising approach to addressing this challenge.

##### Development of open-source software platforms

With the rapid growth of open-source communities, researchers and developers can collaborate and share resources to enhance BCI technology, thereby promoting its widespread application in fields such as healthcare, education, and entertainment. For instance, the MetaBCI open-source platform developed by Tianjin University has received high praise from the research community. Open-source platforms significantly lower technical development barriers and foster interdisciplinary collaboration, accelerating innovation and the popularization of BCI technologies. Through open-source software platforms, users gain greater customization and flexibility, along with enhanced support for data processing, algorithm optimization, and hardware compatibility.

#### Non-invasive EEG acquisition devices

Non-invasive acquisition devices should continue to evolve towards higher throughput. On one hand, there is a need to enable high-resolution EEG signal acquisition to capture more detailed EEG activity. On the other hand, high-density channel devices can provide a more comprehensive mapping of EEG activity, which is crucial for studying brain mechanisms. Additionally, current BCI hardware is generally unacceptable to most healthy individuals [303]. Therefore, there is an urgent need to innovate the electrode design, circuitry, assembly methods, mounting mechanisms, and wearability to make BCI hardwares more compact, comfortable, and user-friendly.

#### Personal privacy safeguard

Taking effective measures to safeguard the privacy of brain data is a pressing issue that demands immediate attention. We will explore several key protective measures to ensure the security and confidentiality of brain data. Firstly, it is imperative to legally protect individuals’ fundamental rights regarding their brain data. Individuals should have the right to refuse forced collection or disclosure of their brain data and to restrict its commercial transfer and use [291]. Secondly, obtaining informed consent is essential for participation in human experimentation. Informed consent protocols should be regularly updated to reflect technological advancements and evolving research practices [291]. Thirdly, brain information should be fully encrypted throughout the entire process, from recording to output, to enhance privacy protection [291]. Finally, the development of BCI applications for AI should prioritize privacy preservation. For instance, these applications should aim to access only the necessary model parameters rather than raw EEG data [306].

#### Other potential frontiers

The future of BCI technologies holds immense development potential, particularly in their deep integration with advanced technologies. Medical rehabilitation is one of the core application areas for BCIs, and based on the latest research developments, we highlight several promising directions for future exploration.

##### Soft robotics

Soft robotics has emerged as a critical area for developing rehabilitation and assistive devices. The inherent compliance of soft robots addresses safety concerns, making them ideal for close human interaction [307]. The softness of these materials allows prosthetic devices to mimic human skin more effectively. Applications of soft robots include tendon-actuated devices, wearable systems, and various assistive devices. Thus, the integration of BCIs with soft robots will greatly broaden the field of research in rehabilitation and assistive technology, becoming a key field of exploration.

##### Haptic sensors and VR

Haptic sensors enhance feedback loops within BCI systems by providing tactile feedback, which is crucial for tasks requiring precision and control [308]. This real-time tactile feedback improves the user experience and enables smoother execution of complex tasks. VR technology dramatically enhances the human–machine interaction experience by creating immersive interactive environments. Users in virtual environments receive rich visual and auditory stimuli, allowing them to focus more effectively during training or therapy [124]. Consequently, combining haptic sensors, VR, and BCIs can significantly expand interaction possibilities.

##### Perceptual reconstruction

Perceptual reconstruction refers to the restoration or substitution of an individual’s lost sensory abilities through technological means. Aging and disease can lead to blindness, depriving patients of the ability to perceive the colorful world. Significant scientific effort has been dedicated to enabling blind individuals to regain sight. Among various approaches, using artificial retinas has faced limitations due to the constrained space within the eye. However, preliminary research suggests that BCIs may offer a promising avenue for treating blindness [309].

## Data Availability

Not applicable.

## Abbreviations

AD: Alzheimer’s disease
AI: Artificial intelligence
ALS: Amyotrophic lateral sclerosis
ATN: Anterior thalamic nuclei
BCIs: Brain-computer interfaces
BIS: Bispectral index
CCA: Canonical correlation analysis
CNNs: Convolutional neural networks
CNS: Central nervous system
CSM: Cerebra state monitor
CSP: Common spatial pattern
DBS: Deep brain stimulation
DCS: Direct current stimulation
DL: Deep learning
ECoG: Electrocorticography
EEG: Electroencephalogram
EEMD: Ensemble empirical mode decomposition
EMG: Electromyographic
EP: Edge paradigm
ERP: Event-related potential
FES: Functional electrical stimulation
fNIRS: Functional near-infrared spectroscopy
GANs: Generative adversarial networks
GCN: Graph convolutional network
HFOs: High-frequency oscillations
HGAD: High generalized anxiety disorder
ICA: Independent component analysis
IMFs: Intrinsic mode functions
LGAD: Low generalized anxiety disorder
LIS: Locked-in syndrome
LSTM: Long short-term memory network
MEA: Microelectrode array
MEG: Magnetoencephalography
MEMD: Multivariate empirical mode decomposition
MEPs: Motor-evoked potentials
MI: Motor imagery
MLP: Multilayer perceptron
MMT: Mammillothalamic tract
fMRI: Functional magnetic resonance imaging
NFB: Neurofeedback
PAC: Phase-amplitude coupling
PD: Parkinson’s disease
PSD: Power spectral density
RNNs: Recurrent neural networks
SCI: Spinal cord injury
SSC: Sleep stage classification
SSVEP: Steady-state visual evoked potentials
tDCS: Transcranial DCS
TSCNN: Two-stream convolutional neural network
VNS: Vagus nerve stimulator
VR: Virtual reality
WT: Wavelet transform
